# Pkcδ Activation is Involved in ROS-Mediated Mitochondrial Dysfunction and Apoptosis in Cardiomyocytes Exposed to Advanced Glycation End Products (Ages)

**DOI:** 10.14336/AD.2017.0924

**Published:** 2018-08-01

**Authors:** Yao-Chih Yang, Cheng-Yen Tsai, Chien-Lin Chen, Chia-Hua Kuo, Chien-Wen Hou, Shi-Yann Cheng, Ritu Aneja, Chih-Yang Huang, Wei-Wen Kuo

**Affiliations:** ^1^Department of Biological Science and Technology, College of Biopharmaceutical and Food Sciences, China Medical University, Taiwan.; ^2^Department of Pediatrics, China Medical University Beigang Hospital, Taiwan.; ^3^School of Chinese Medicine, College of Chinese Medicine, China Medical University, Taiwan.; ^4^Department of Life Sciences, National Chung Hsing University, Taiwan.; ^5^Laboratory of Exercise Biochemistry, University of Taipei, Taipei, Taiwan.; ^6^Graduate Institute of Physical Therapy and Rehabilitation Science, China Medical University, Taiwan.; ^7^Department of Medical Education and Research and Department of Obstetrics and Gynecology, China Medical University Beigang Hospital, Taiwan.; ^8^Department of Obstetrics and Gynecology, China Medical University An Nan Hospital, Taiwan.; ^9^Obstetrics and Gynecology, School of Medicine, China Medical University, Taichung, Taiwan.; ^10^Department of Biology, Georgia State University, Atlanta, GA 30303, USA.; ^11^Graduate Institute of Basic Medical Science, China Medical University, Taichung, Taiwan; Graduate Institute of Chinese Medical Science, School of Chinese Medicine, China Medical University, Taichung, Taiwan; Department of Health and Nutrition Biotechnology, Asia University, Taichung, Taiwan.

**Keywords:** advanced glycation end products (AGEs), apoptosis, diabetes mellitus (DM), mitochondrial, protein kinase C (PKC)δ, reactive oxygen species (ROS)

## Abstract

Diabetic patients exhibit serum AGE accumulation, which is associated with reactive oxygen species (ROS) production and diabetic cardiomyopathy. ROS-induced PKCδ activation is linked to mitochondrial dysfunction in human cells. However, the role of PKCδ in cardiac and mitochondrial dysfunction caused by AGE in diabetes is still unclear. AGE-BSA-treated cardiac cells showed dose- and time-dependent cell apoptosis, ROS generation, and selective PKCδ activation, which were reversed by NAC and rotenone. Similar tendency was also observed in diabetic and obese animal hearts. Furthermore, enhanced apoptosis and reduced survival signaling by AGE-BSA or PKCδ-WT transfection were reversed by kinase-deficient (KD) of PKCδ transfection or PKCδ inhibitor, respectively, indicating that AGE-BSA-induced cardiomyocyte death is PKCδ-dependent. Increased levels of mitochondrial mass as well as mitochondrial fission by AGE-BSA or PKCδ activator were reduced by rottlerin, siPKCδ or KD transfection, indicating that the AGE-BSA-induced mitochondrial damage is PKCδ-dependent. Using super-resolution microscopy, we confirmed that PKCδ colocalized with mitochondria. Interestingly, the mitochondrial functional analysis by Seahorse XF-24 flux analyzer showed similar results. Our findings indicated that cardiac PKCδ activation mediates AGE-BSA-induced cardiomyocyte apoptosis via ROS production and may play a key role in the development of cardiac mitochondrial dysfunction in rats with diabetes and obesity.

Continuous hyperglycemia, a common feature of both type I and type II diabetes mellitus (DM) patients, plays a crucial role in diabetic complications in macrovessel and microvessel structures. A particular target organ in this setting is the heart. The destruction of cardiac function has been well recognized as a result of cardiomyocyte apoptosis in both clinical and experimental diabetes [1-3]. Numerous studies have reported that a direct correlation between the pathogenesis of diabetic heart disease and the reactive oxygen species (ROS) production, which is induced by hyperglycemia-derived advanced glycation end products (AGEs) [4, 5]. Therefore, the mechanism through which AGE formation induced by high glucose exposure stimulates ROS generation to cause cardiomyocyte apoptosis needs to be elucidated [6].

AGEs are a heterogeneous group of compounds produced from the non-enzymatic glycation or glycoxidation of proteins, lipids and nucleic acids with carbonyl methylglyoxal (MGO), a major AGE precursor. AGE formation is directly accelerated in chronic high glucose condition and is associated with an increased generation of ROS [7, 8]. Although AGEs in plasma and tissues accumulate slowly during natural aging, they are dramatically elevated in patients with DM, contributing to the development of diabetic complications. In addition, through the association with the receptor for AGE (RAGE), the accumulated AGEs can initiate a cascade of signal transduction to activate several worse outcomes, such as oxidative stress, inflammation and apoptosis, leading to target cell dysfunction [7, 8]. Our previous studies [5, 9, 10] using experimental diabetic rat models showed that hyperglycemia can cause reduced cardiac contraction function evidenced by the decreased ejection fraction and fraction shortening. However, the signaling involved in hyperglycemia-derived AGEs induced cardiac apoptosis need to be further identified.

Protein kinase Cδ (PKCδ), a member of the novel PKC subfamily, is involved in cell apoptosis in a stimulus- and tissue-specific manner; it regulates the expression and function of apoptotic-related proteins and is itself a target for caspases [11-13]. Activation of PKCδ by various apoptotic stimuli results in the translocation of PKCδ to distinct cellular compartments. For example, PKCδ activation during reperfusion results in translocation to the mitochondria where it can negatively regulate mitochondrial function and initiate pro-apoptotic pathways by direct effects on mitochondria [14-16]. Additionally, it is reported that PKCδ is phosphorylated in cells treated with H_2_O_2_ [17]. Moreover, H_2_O_2_-induced apoptosis is blocked by the inhibition of PKC activation and translocation to the mitochondria [18], indicating that ROS-induced cell apoptosis may be dependent on PKCδ activation and mitochondria translocation.

It has been reported that aberrant mitochondrial dynamic and degeneration in cardiac disorder induced by high glucose-derived ROS is associated with cardiomyocyte apoptosis [19]. The PKCδ activation is involved in ROS-mediated mitochondrial dysregulation [20]. Because the hearts are particularly responsive to the changes in mitochondria function due to their high energy demands [19], the triggers for this cardiac mitochondrial dysfunction to further cause cell death are needed to be revealed. Therefore, here is the study to identify whether ROS-mediated PKCδ activation is involved in AGE-induced cardiac mitochondrial dysfunction and cell apoptosis to cause heart failure.

Actuarially, several studies have identified the involvement of PKC isoforms in pathogenesis of heart disease, including diabetic cardiomyopathy [21, 22]. However, very little work focused on the association of PKCδ activated mitochondrial dysregulation with heart diseases, such as diabetic cardiac disorder. In this study, we investigated the role of PKCδ in AGE-induced cardiac apoptosis *in vitro* and *in vivo*. We found that PKCδ was dose-dependently activated in cardiomyocytes administered with AGEs, and AGEs also induced cardiac mitochondrial fission and cell apoptosis. We identified a regulatory pathway for AGE-induced apoptosis through PKCδ activation. The results on PKCδ activation suggest a target to protect cardiomyocytes against AGE-induced apoptosis for the treatment of diabetic cardiomyopathy.

## MATERIAL AND METHODS

### Cell culture

H9c2 cardiomyoblast cells derived from embryonic BD1X rat heart tissue were obtained from American Type Culture Collection (ATCC^®^ CRL-1446™, Manassas, VA, USA), and cultured in Dulbecco’s modified eagle’s medium (DMEM, 12100-046; Gibco, Grand Island, NY, USA) with 10% fetal bovine serum (FBS; Hyclone, U.S.), Penicillin-Streptomycin (Pen/Strep, 15140122; Gibco BRL, Paisley, Scotland, USA) in a humidified atmosphere containing 5% CO_2_ at 37 °C. Experiments on H9c2 cells were performed with 80% confluent cells that had been seeded in 100-mm.

### Neonatal rat ventricular myocytes primary culture

NRVMs were prepared and cultured using a Neonatal Rat/Mouse Cardiomyocyte Isolation Kit (nc-6031; Cellutron Life Technology, Baltimore, MD, USA) which was previously described [23]. Hearts were dissected from 1- to 3-day-old Sprague-Dawley rats and transferred into a sterile beaker. Each heart was digested and stirred in the beaker at 37?°C for 12?min. The supernatant was then transferred to a new sterile tube and spun at 1200?r.p.m. for 1?min. The cell pellets were then re-suspended in D3 buffer and pre-plated for 1?h by seeding on an uncoated plate at 37?°C in a CO_2_ incubator to select the cardiac fibroblasts. The unattached cells were transferred onto plates that were precoated with NS medium (supplemented with 10% fetal bovine serum). After overnight culture, the NS medium was replaced with a serum-free NW (without serum) medium. The cardiomyocyte cultures were ready for experiments 48?h after the initial plating.

### Glucose derived-AGE preparation

AGEs were prepared according to methods described previously [24-26]. AGE was prepared by incubating bovine serum albumin (A8806, BSA, fraction V, fatty acid-free, endotoxin-free; Sigma Chemical, 100 mg/mL) with D-glucose (1 M) in dulbecco’s phosphate buffered saline (DPBS, 21600-010; Gibco) for 12 weeks at 37 °C. Unmodified BSA was prepared under the same conditions without glucose as a control. Fluorescence of the supernatant was determined (excitation 370 nm, emission 440 nm) using LJL Biosystems (AnalystTM AD, Sunnyvale, CA, USA), which confirmed the higher intensity of AGE in AGE-modified BSA than that in unmodified BSA (Suppl. Fig. S10). The AGE solution was filtered to be sterile by 0.8 μM Millex GP filter unit (Millipore, Billerica, MA, USA).

### MTT assay for cell viability

Cell viability was measured using the MTT assay as previously described [27], and based on the 3-(4, 5-Dimethylthiazol-2-yl)-2, 5-diphenyltetrazolium bromide (MTT, M2003; Sigma, St. Louis, MO, USA) conversion into formazan crystals using mitochondrial dehydrogenases. The prepared H9c2 cells suspension was seeded into 96-well plates at a density of 1 × 10^4^ cells/well and incubated at 37 °C for 24 h. Then the cells were treated with (0-300 μg/ml) AGEs as indicated for (0-36 h). The supernatant was discarded and the culture medium was replaced with 100 μl of MTT solution (5 mg/ml stock solution in DPBS, diluted with culture medium to the final concentration 0.5 mg/ml) was added into each well. Then the cells were maintained at 37 °C for 4 h, this solution was removed and the formed formazan was dissolved with 100μl dimethyl sulfoxide (DMSO). The optical density (O.D.) values of samples were read at 580 nm with a micro plate reader.

### Reactive oxygen species production

Intracellular generation of ROS was examined by peroxide-sensitive fluorescent probe 2′, 7′-dichloro-fluorescein diacetate (CM-H2 DCFH-DA, C6827; Molecular Probes, Invitrogen, Carlsbad, CA, USA) as previously described [10], the fluorescent dichloro-fluorescein (DCF) formed by oxidation of DCFH was quantified by flow cytometry. Briefly, cells were incubated with (5 μM) CM-H2 DCFDA for 30 min at 37 °C in dark. The following inhibitors were used the ROS scavenger, N-acetyl-L-cysteine (NAC, 500 μM, Sigma, St. Louis, MO, USA); the Mitochondrial Complex I inhibitor Rotenone (0.1μM, Sigma, St. Louis, MO, USA). Additionally, mitochondrial superoxide (O_2_ radical dot^-^) was evaluated by MitoSOX™ Red mitochondrial superoxide indicator (M36008, Molecular Probes, Invitrogen, Eugene, OR, USA). Cells were resuspended with MitoSOX™ (2 μM) and allowed to incubate at 37 °C for 10-15 min. Samples were analyzed by a BD FACSCantoM II flow cytometer (Becton-Dickinson, Franklin Lakes, NJ) to identify mean mitochondrial ROS production.

### Transfection of plasmid DNA and siRNA assay

Cells with 50% confluence were replaced into fresh culture medium containing serum then plasmid of pEGFP N1-PKCδ (GFP-PKCδ-WT) and pEGFP N1-PKCδ^K376R^ (GFP-PKCδ-KD; PKCδ kinase dominant negative mutant) which were obtained from Department of Life Science and the Graduate Institute of Biomedical Sciences, National Chung Hsing University, Dr. H.C. Chen lab [28], were transfected in the cells AGE or not for 24 hours using PureFection™ Nanotechnology-based Transfection Reagent (System Biosciences, Inc., Mountain View, CA, USA) following the manufacture protocol. Double-stranded si-RNA sequences targeting PKCδ mRNAs were obtained from EMD Millipore Corporation. Then non-specific si-RNA (scramble) consisted of a non-targeting double-stranded RNA. Cells were cultured in 100-mm plates in medium. Transfection of si-RNA was carried out with transfection reagent. Specific silencing was confirmed by immunoblotting with cellular extracts after transfection.

### Determination of mitochondrial mass

Mitochondrial mass was measured with the fluorescent dye nonyl acridine orange (NAO, Molecular Probes; Invitrogen, Eugene, OR, USA), which binds to cardiolipin in mitochondrial inner membrane. The cells at subconfluent stage were trypsinized and resuspended in 0.5 ml of DPBS containing 2.5 μM NAO. After incubation for 15 min at 25°C in the dark, cells were immediately transferred to a tube for analysis on a flow cytometry (Becton-Dickinson FACSCanto, Franklin Lakes, NJ). The excitation wavelength was set at 488 nm and the intensity of emitted fluorescence of a total of 10,000 cells at 525 nm was recorded on channel FL1.

### Animal model and treatments

Male Wistar rats (four-week old) were purchased from the National Animal Breeding and Research Center (Taipei, Taiwan). All the animals were maintained under a 12-h light/dark cycle at a constant temperature (25 °C) and were provided access to water and standard laboratory chow (Lab Diet 5001; PMI Nutrition International Inc., Brentwood, MO, USA). Room conditions and experimental procedures were followed according to the NIH Guide for the Care and Use of Laboratory Animals and all protocols were approved by the Institutional Animal Care and Use Committee of China Medical University, Taichung, Taiwan. The animals were arranged into two groups (n = 5 each): Wistar rats, Wistar rats received of intraperitoneal (IP) streptozotocin (STZ; Sigma, St. Louis, MO). After one week of acclimatization, diabetes was induced by the injecting STZ (65 mg/kg body weight in a citrate buffer, pH 4.5) into a lateral tail vein. After three days of injection, glucose level was measured with the Accu-Check Compact kit (Roche Diagnostics Gmbh, Mannheim, Germany). Sixteen days after treatment the animals were sacrificed and hearts were removed for further analysis. All 12 male SD hamsters (eight weeks old, 300 g weight) were purchased from BioLASCO Taiwan Co., Ltd. (Taipei, Taiwan) and divided into two groups (n = 6 each). The control hamster group was labeled as control. The high-fat diet treatment hamster group was labeled as HFD.

### Western blot analysis

The protein levels of cultured cells were performed as previously described [27]. Cultured H9c2 cell were scraped and washed twice with DPBS, then cell suspension was spun down, and cell pellets were lysed for 30min in lysis buffer (50mM Tris (pH 7.5), 0.5M NaCl, 1.0mM EDTA (pH 7.5), 10% glycerol. Spin down in 12,000 rpm for 30 minutes then the supernatant was collected. Protein samples were separated in 8~10% SDS-PAGE and transferred to polyvinylidene difluoride (PVDF; Millipore, Billerica, MA, USA) membranes. Nonspecific protein binding was prohibited by a blocking buffer (5% milk, 20 mM pH 7.6 Tris-HCl, 150 mM NaCl, and 0.1% Tween-20) and the proteins were blotted with specific antibodies in the blocking buffer at 4 °C overnight. After incubations with a secondary antibody for 2 h, the densitometry of immunoblots was analyzed by Fuji LAS 3000 imaging system. Primary antibodies PKCδ, θ, ζ, Caspase-3, Cleaved Caspase-3, Caspase-9, Cleaved Caspase-9, COX IV, Phospho-DRP1, Phospho-PKC θ, ζ, p62/SQSTM1, LC3B (#2058, #2059, #9368, #9665s, #9664, #9506, #9507, #11967, 3455s, #9377, #2060, #5114, #2775, Cell signaling), PKCα, Phosphor- PKCα (05-154, 06-822, Upstate), Phospho-PKCδ T505/507 (#9374, Cell signaling, NBP1-51405, Novus, bs-3727R, Bioss), β-Actin, Phospho-Akt, Bcl2, Bax, Bcl_XL_ Bak, Cytochrome *C*,DRP1, GFP, Parkin, PINK1 (sc-47778, sc-7985, sc-7382, sc-526, sc-8392, sc-7873, sc-13560, sc-32898, sc-8334, sc- 30130, sc-33796, Santa Cruz).

### Immunohistochemistry

The examination of PKCδ protein in cardiac tissues was performed as previously described [29]. The tissue sections were deparaffinized and rehydrated. After three washes in DPBS, we then used 1% bovine serum albumin to block nonspecific binding. Then the sections were incubated with primary antibody against PKCδ (1:100 dilution; bs-3727R; Bioss) at 4°C overnight in a moist chamber. Blocking solution without the primary antibody was used as a negative control. After being incubated with horseradish peroxidase for 30 minutes at 37°C, the slides were incubated with 3,3′-diaminobenzidine (DAB; Sigma) solution for visualization. After washing by PBS fir 10 minutes the tissue microarray was then detected by using microscopy (magnification: 200X) (Olympus, Tokyo japan). Primary antibody Phospho-PKCδ T505/507 (bs-3727R; Bioss).

### Indirect immunofluorescence and confocal microscopy

We carried out mitochondrial staining as described [30]. Expression of phosphorylation PKCδ protein was analyzed on cells with a Leica TCS SP2 confocal microscope using a monoclonal Phospho-PKCδ Thr505/507 (bs-3727R, Bioss) antibody. Briefly, we plated the cells (1×10^4^) onto the chamber slide. After treatment, we stained them for 20 min with 0.02 μM MitoTracker Red CMXRos (M7510; Molecular probes, Invitrogen, Grand Island, NY, USA). Cells were fixed with 4% paraformaldehyde (Sigma-Aldrich, St. Louis, MO) for 15?min at room temperature and permeabilized with 0.1% Triton X-100 (Merck, Germany) for 15?min at room temperature before staining with a specific antibody. Then, the cells were washed and stained with Alexa 546 rabbit anti-mouse IgG secondary antibodies (A-11060; Invitrogen, Carlsbad, CA, USA) for 2 hours at 37?, and rinsed and mounted. The cellular nuclei were stained by 4′, 6-diamidino-2-phenylindole (DAPI; Sigma, St. Louis, MO, USA). Images were captured using a Leica SP2 Confocal Spectral Microscope (Buffalo Grove, IL, USA). The images were processed using Adobe Photoshop.

### N-SIM: Nikon-structured illumination microscope

Samples were essentially prepared as before the confocal experiment. Super-resolution imaging was performed with a Nikon-structured illumination microscope (N-SIM) system equipped with a CFI Plan Apochromat IR 60x WI DIC N2 (NA 1.27) immersion objective and an iXon3 camera (DU-897 X-5472, Andor Technology). 3D-SIM image stacks were acquired with a Z-distance of 0.2 μm, covering the entire thickness of the cell (about 3.1 μm). The camera settings were configured as follows: format for capture, no binning; exposure time, 150 ms (1 frame recommended); readout mode, EM gain 10 MHz 14-bit; gain multiplier, 20-200 ms (max 300); conversion gain. 5.1× 15 raw images per plane were acquired and computationally reconstructed using the reconstruction slice system from NIS-Elements software (Nikon).

### Immunoprecipitation

Immunoprecipitations were performed using H9c2 cell lysates and the PureProteome Protein G Magnetic Bead System (Millipore) according to the manufacturer’s instructions. The total cell lysates (500µg) for each sample were specifically bond with 5?µg of a specific primary antibody, and the mixture was incubated on a rotator at 4°C overnight. The immunoprecipitates were collected by centrifugation at 2,500?rpm for 5?min at 4°C, supernatants were discarded and pellet was resuspended with 2× electrophoresis sample buffer by boiling at 95°C for 10?min and separated by SDS-PAGE.

### Mitochondria isolation

Mitochondria were isolated using a mitochondria isolation kit (Thermo Scientific, Rockford, IL, USA) according to the manufacturer’s instructions. Mitochondrial subpopulation pellets were resuspended in sucrose-based SEM buffer (250 mM sucrose, 1 mM EDTA, and 10 mM MOPS, pH 7.2) depending on the assay to be performed. Mitochondrial protein concentrations were determined using the Bradford method as a standard, and analyzed by western blotting.

### Measurement of bioenergetic parameters

To determine cellular oxygen consumption rate (OCR) and extracellular acidification rate (ECAR) of the primary culture of NRVMs and H9c2, we used the XF24 Analyzer (Seahorse Bioscience, North Billerica, MA, USA). Briefly, a seeding density of 1.5×10^4^ cells per well in 200-500 μl growth medium on an XF24-well microplate (Seahorse Bioscience) were adhered and incubated at 37 °C, then after treatment the culture medium was replaced 1 h prior to measurement by the assay medium pre-warmed to 37°C (pH 7.4). The program of Seahorse XF24 Analyzer was set according to the manufacturer’s recommendation and the data are expressed in pmol/min/10^4^ cells for OCR and in mpH/min/10^4^ cells for ECAR to allow comparison between independent experiments. The OCR and ECAR reflect the metabolic activities of the cells and the numbers of cells, therefore the values were normalized to the total amount of cells in each well.

### Statistical analysis

All experiments data were performed from three individual experiments and presented as mean ± S.E.M. One-way ANOVA was performed to compare the statistical difference of all groups. The level of *p* < 0.05 was considered statistically significant.

## RESULTS

### Effects of AGE-BSA treatment on cell viability, apoptosis and ROS generation

**Figure 1.**
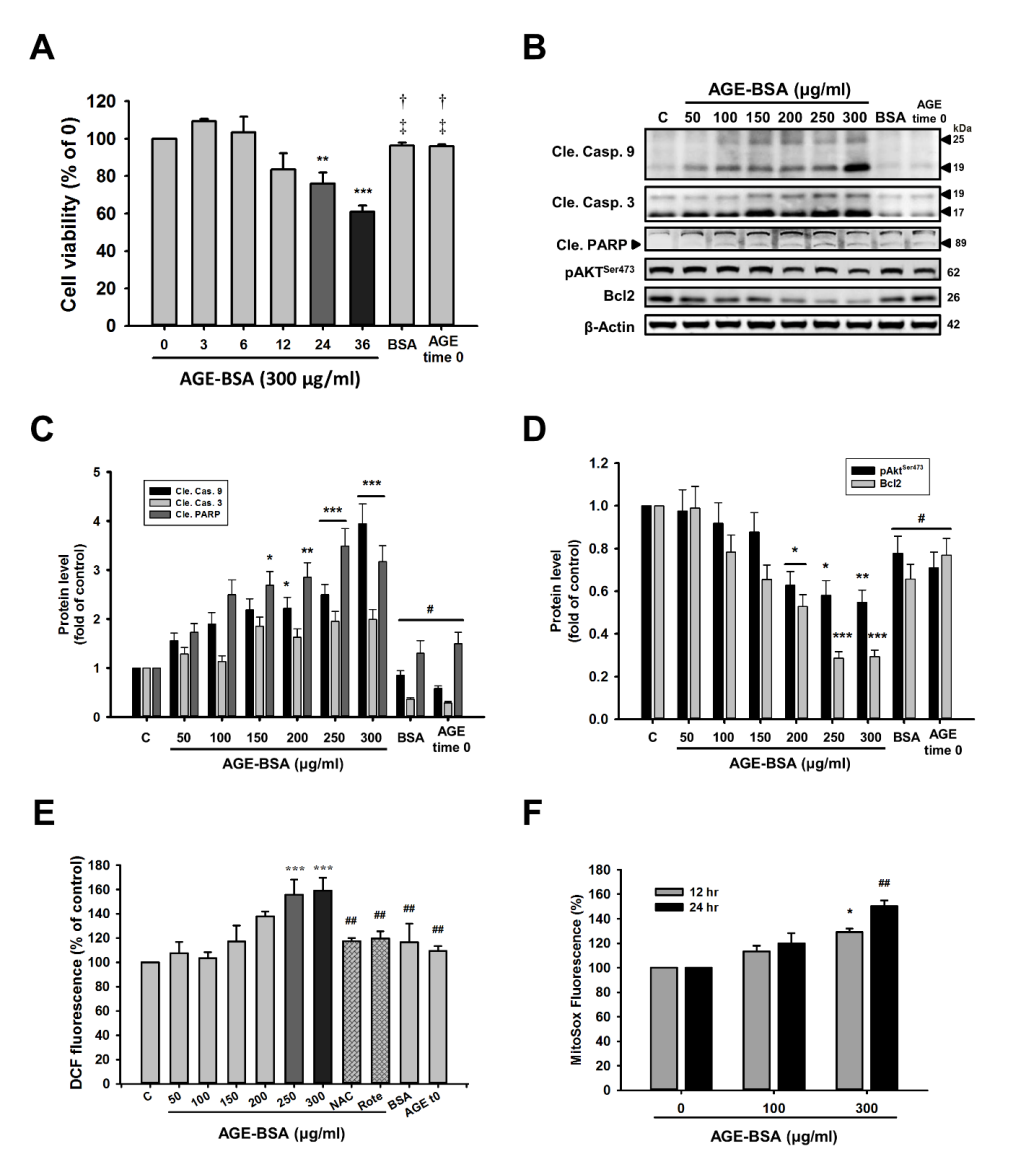
AGE-BSA reduces cell viability, enhanced apoptosis and ROS generation in H9c2 cells in a time- and dose-dependent manner (**A**) Cells were treated with 300 μg/ml of AGE-BSA for different time periods as indicated or non-glycated BSA. Cell viability was determined using MTT assays. (**B**) Cells were treated with AGE-BSA at different concentrations as indicated. Levels of apoptosis-related proteins were analyzed by western blotting. These are cropped blots; full-length blots are presented in Suppl. Figure S1. (**C, D**) Bar graphs show relative optical densities of the (fig. 1B) apoptosis and survival protein levels at 24 h. (**E**) Intracellular ROS levels of fluorescence intensities of DCF and (**F**) the mitochondrial ROS levels of AGE-BSA-exposed cardiac cells at the indicated doses and time periods were examined by flow cytometry. NAC (500 μM); rotenone (Rote) (0.1 μM). Bars indicate the mean ± SEM obtained from experiments performed in triplicate. ^*^P<0.05, ^**^P <0.01 and ^***^P<0.001 compared with the control group; *^#^P*<0.05 and ^##^*P*<0.01compared with the 300 μg/mg group; *^†^P*<0.05 when compared with 24 h or 36 h.

Cells were cultured with AGE-BSA or non-glycated BSA, and cell viability was determined at 3, 6, 12, 24, 36 h after AGE exposure (Fig 1A). AGE-BSA time-dependently decreased cell viability compared to control at 24 h. However, treatment with bovine serum albumin (BSA) and AGE-BSA time 0 (T0) did not significantly change cell growth (*P* >0.05). The analysis results of apoptotic proteins as shown in Fig. 1(B & C) (Suppl. Fig. S1), AGE-BSA significantly increased cleaved caspase-9, cleaved caspase-3 and cleaved PARP levels. In contrast, AGE-BSA markedly reduced Akt phosphorylation and Bcl-2 survival protein levels (Fig. 1D). These data suggest that AGE-BSA-induced cell apoptosis was associated with proteins related to mitochondria-dependent apoptosis (Suppl. Fig. S9).

To examine the ROS production, the mean fluorescence intensities (MFIs) were measured using 2′,7′-Dichlorofluorescin diacetate (DCFH-DA) and mitochondrial superoxide (MitoSox) Red. As shown in Fig. 1E (Suppl. Fig. S8), cardiac cells dose-dependently increased cellular ROS production compared to control. This increase was attenuated by N-acetyl-L-cysteine (NAC) (500 μM) and the mitochondrial complex I inhibitor, rotenone (Rote) (0.1 μM).

As shown in Fig. 1(F), after treatment with 100 or 300 μg/ml AGE-BSA for 12 or 24 h, mitochondrial ROS production was increased in a dose- and time-dependent manner. These results indicated that AGE-BSA significantly increased intracellular ROS and mitochondrial-derived superoxide generation.

**Figure 2.**
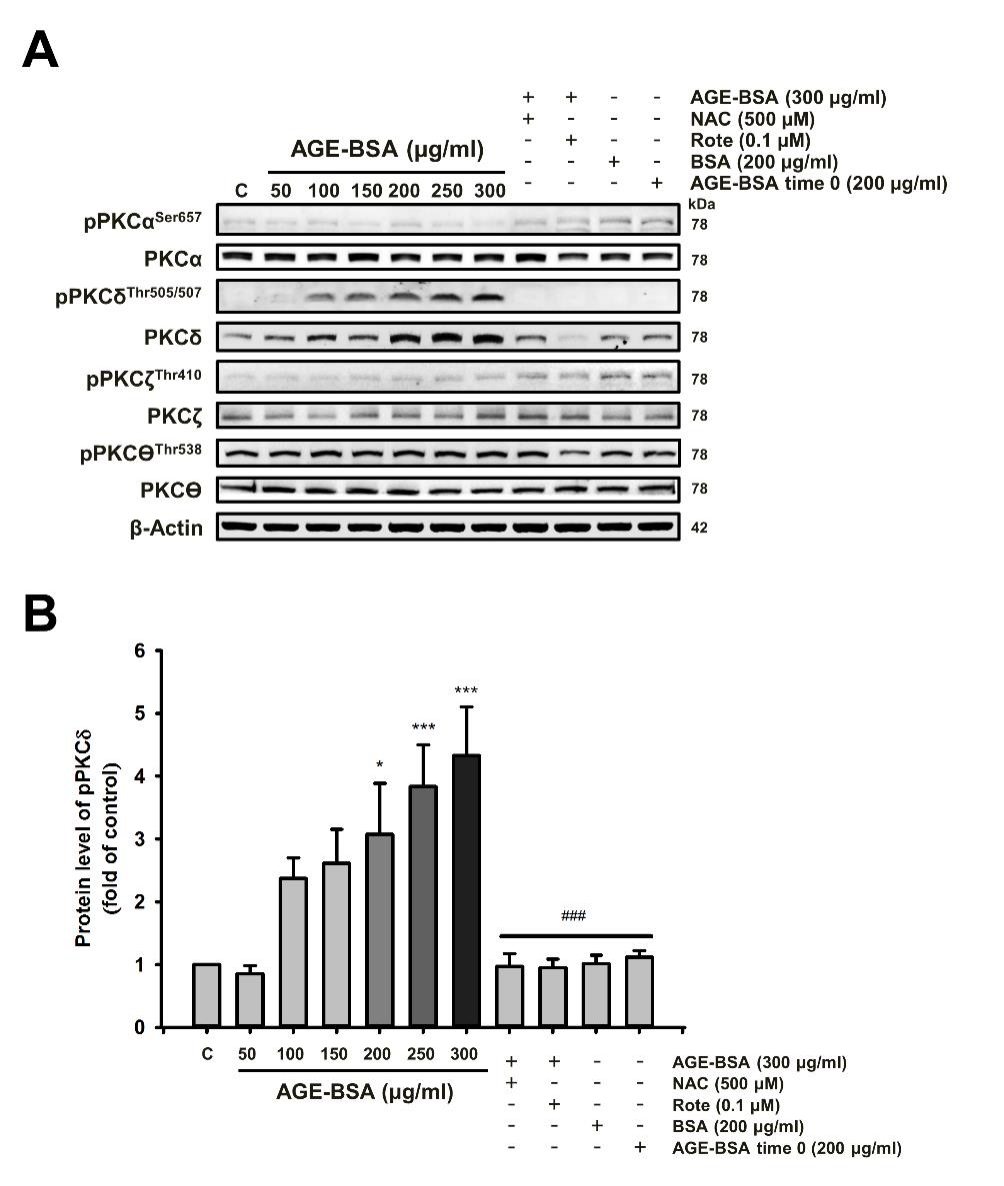
Among PKC isoforms, AGE-BSA specifically induces PKCδ protein expression and phosphorylation in cardiac cells (**A**) Cells were treated with different doses of AGE-BSA with or without ROS scavengers (NAC) and mitochondrial complex I inhibitor (Rote) as indicated for 24 h. Western blot analyses were performed with antibodies against the PKC isoforms. These are cropped blots; full-length blots are presented in Suppl. Figure S2. (**B**) The densitometry measurements show the quantitative results of the western blots. Bars indicate the mean ± SEM obtained from experiments performed in triplicate. ^*^P<0.05 and ^***^P<0.001 compared with the control group; ^###^*P*<0.001 compared with the AGE-BSA (300 μg/ml) group.

### Cardiac PKCδ expression and phosphorylation and apoptosis-related proteins in diseased animal models with elevated AGE levels

To investigate whether the AGE-BSA can induce the PKCδ and PKCδ phosphorylation which is mediated through ROS, cells were treated with different doses of AGE-BSA, and western blotting was performed. As shown in Fig. 2(A & 2B) (Suppl. Fig. S2), the results demonstrated that the total and phosphorylated protein levels of PKCδ rather than other isoforms were dose-dependently increased in cardiac cells treated with AGE-BSA (0-300 μM) for 24 h. This increase was reduced by the treatments of NAC and rotenone.

**Figure 3.**
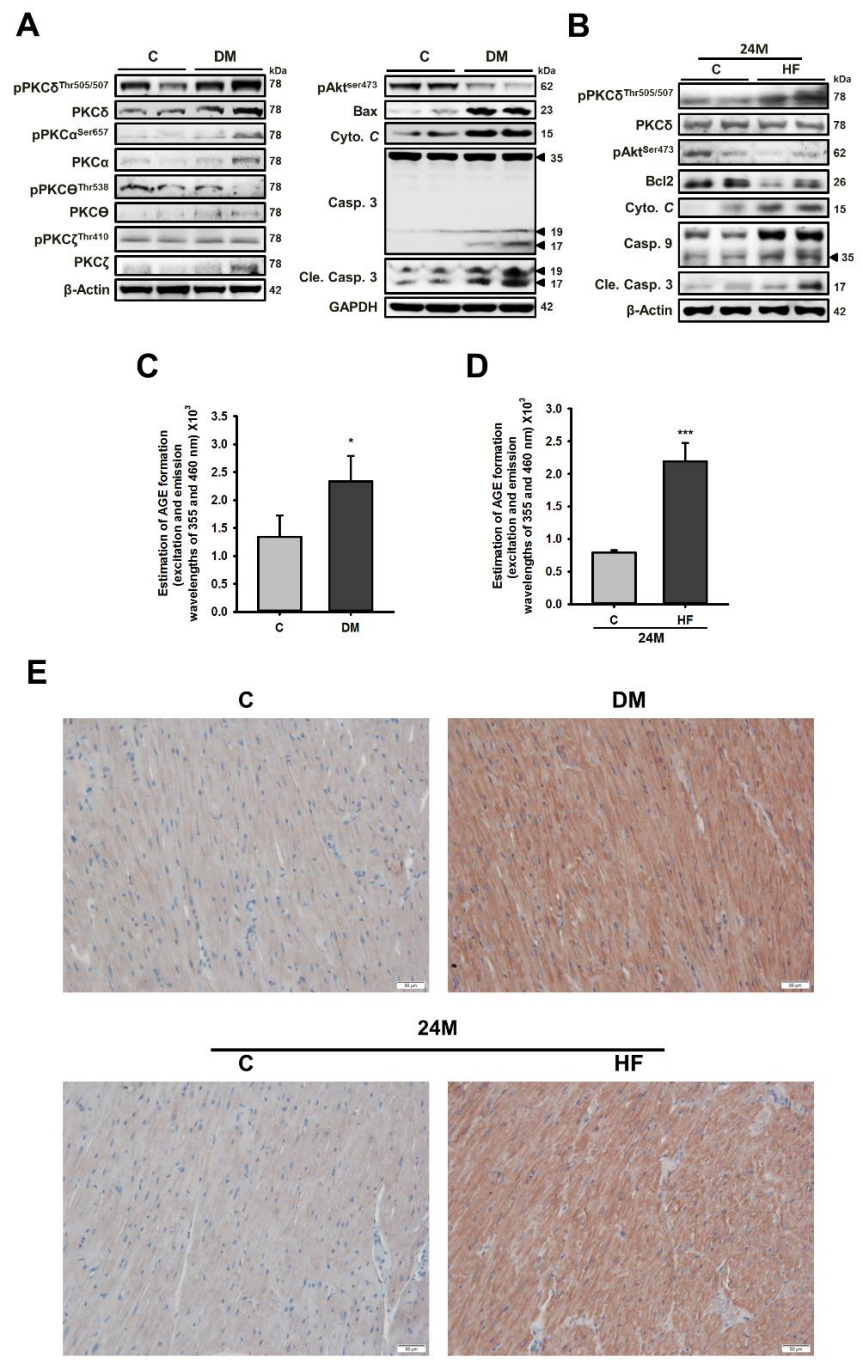
Cardiac PKCδ expression and phosphorylation as well as apoptosis-related proteins are increased in diseased animal models with elevated circulatory AGE levels Western blot analysis of the cardiac expression and phosphorylation levels of PKC isoforms and apoptosis-related proteins in rats with (**A**) diabetes mellitus (DM) and (**B**) a high-fat (HF) diet. Serum AGE levels in rats with (**C**) DM; (**D**) HF diet. These are cropped blots; full-length blots are presented in Suppl. Figure S3. Protocols for animal models with DM and HF diets and serum AGE analysis were described in the methods section. (**E**) Cardiac expression of phosphorylated PKCδ was examined by immunohistochemistry analysis. Bars indicate the mean ± SEM obtained from experiments performed in triplicate. ^*^P<0.05 and ^***^P<0.001 compared with the control group. DM, Diabetes mellitus; HF, high-fat diet.

To determine the expression profile of PKC family proteins in diseased animal hearts, the protein levels were analyzed and the results showed that cardiac PKCδ and its phosphrylation rather than other PKC isoforms were induced in the diabetic and aging high-fat (HF) diet-treated rat hearts (Fig. 3A & B) (Suppl. Fig. S3). Furthermore, an increase in apoptotic protein indicates that AGE-BSA induced apoptosis in AGE-BSA exposed cells (Fig. 1B). Western blot analysis of left ventricles from Diabetes mellitus (DM) rats and high-fat (HF) hamsters revealed a similar trend in p-Akt, cytochrome *C* and caspase-3 levels, indicating the development of apoptosis associated with cardiac dysfunction. The echo cardiographic analysis of experimental diabetic hearts demonstrated the reduced cardiac function evidenced by decreased ejection fraction and fraction shortening (Data was shown in our previous studies [5, 9, 10].) The serum AGE levels were measured with a spectrometer and increased in diseased animals (Fig 3C & 3D). As shown in Fig. 3(E), we also characterized AGE-BSA-exposed cells using Immunohistochemistry (IHC) with antibodies against pPKCδ Threonine (Thr) 505/507, which was significantly higher in diseased animal hearts. These results were consistent with those from western blotting.

**Figure 4.**
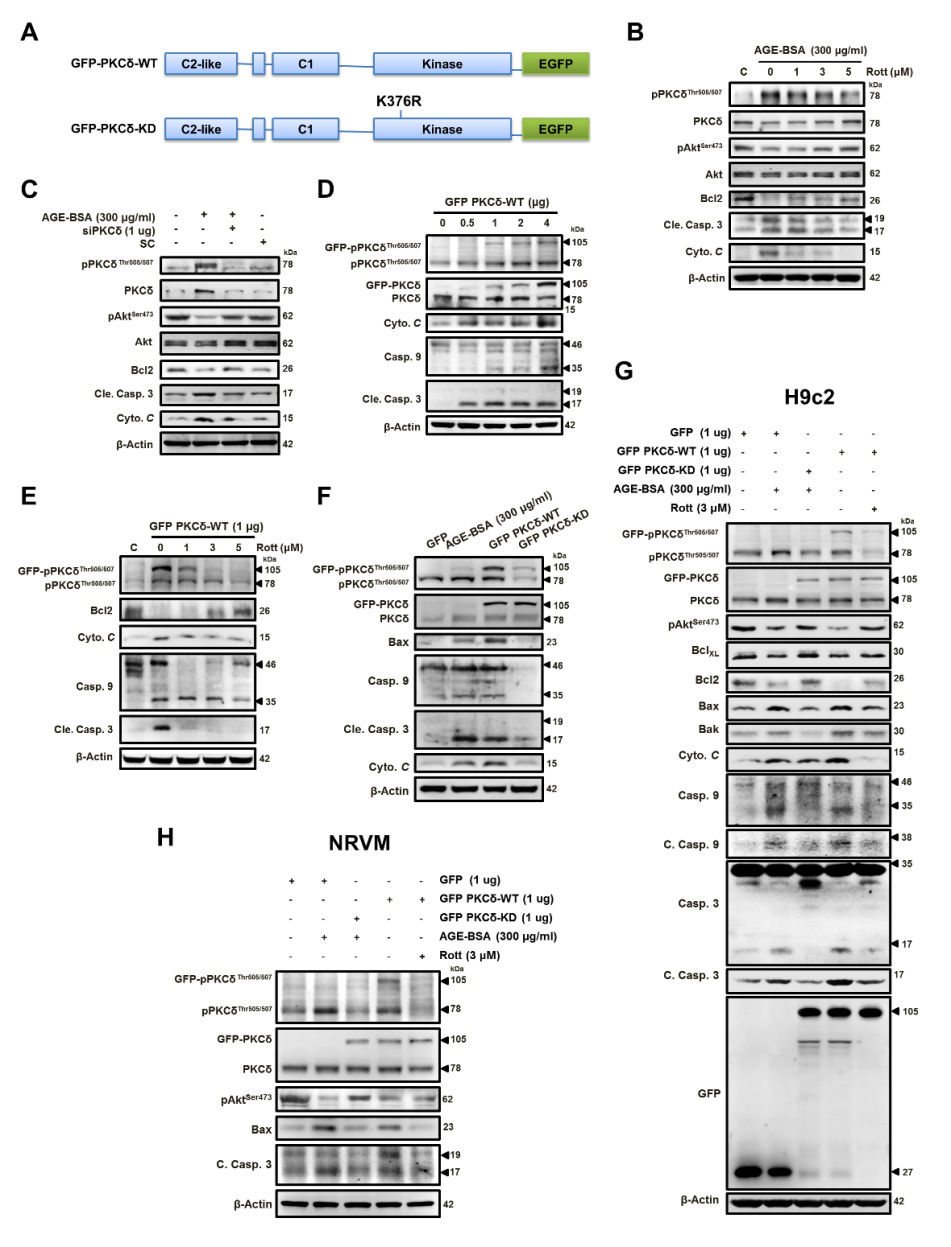
AGE-BSA-induced cardiomyocyte apoptosis is mediated through PKCδ activation. (**A**) The diagram depicts the domain organization of GFP-PKCδ. The GFP-PKCδ derivatives, including the wild-type (WT) and the kinase-deficient mutant (KD; K376R). Cells were treated with AGE-BSA (300 μg/ml) and (**B**) rottlerin (1-5 μM) or (**C**) PKCδ silencing. (**D**) Cells were transfected with GFP-fused PKCδ (GFP PKCδ-WT) at different doses as indicated and with (**E**) 1 μg rottlerin (1-5 μM). (**F&G**) H9c2 cells or (**H**) neonatal rat ventricular myocytes (NRVM) were exposed to AGEs (300 μg/ml) with or without (GFP PKCδ-KD) transfection or transfected with GFP PKCδ-WT in the presence of rottlerin (3 μM) or not. These are cropped blots, full-length blots of PKCδ and pPKCδ are presented in Suppl. Figure S4. SC, scramble; WT, wild type; KD, kinase-deficient; All the proteins were analyzed by western blotting using β-actin as a loading control.

**Figure 5.**
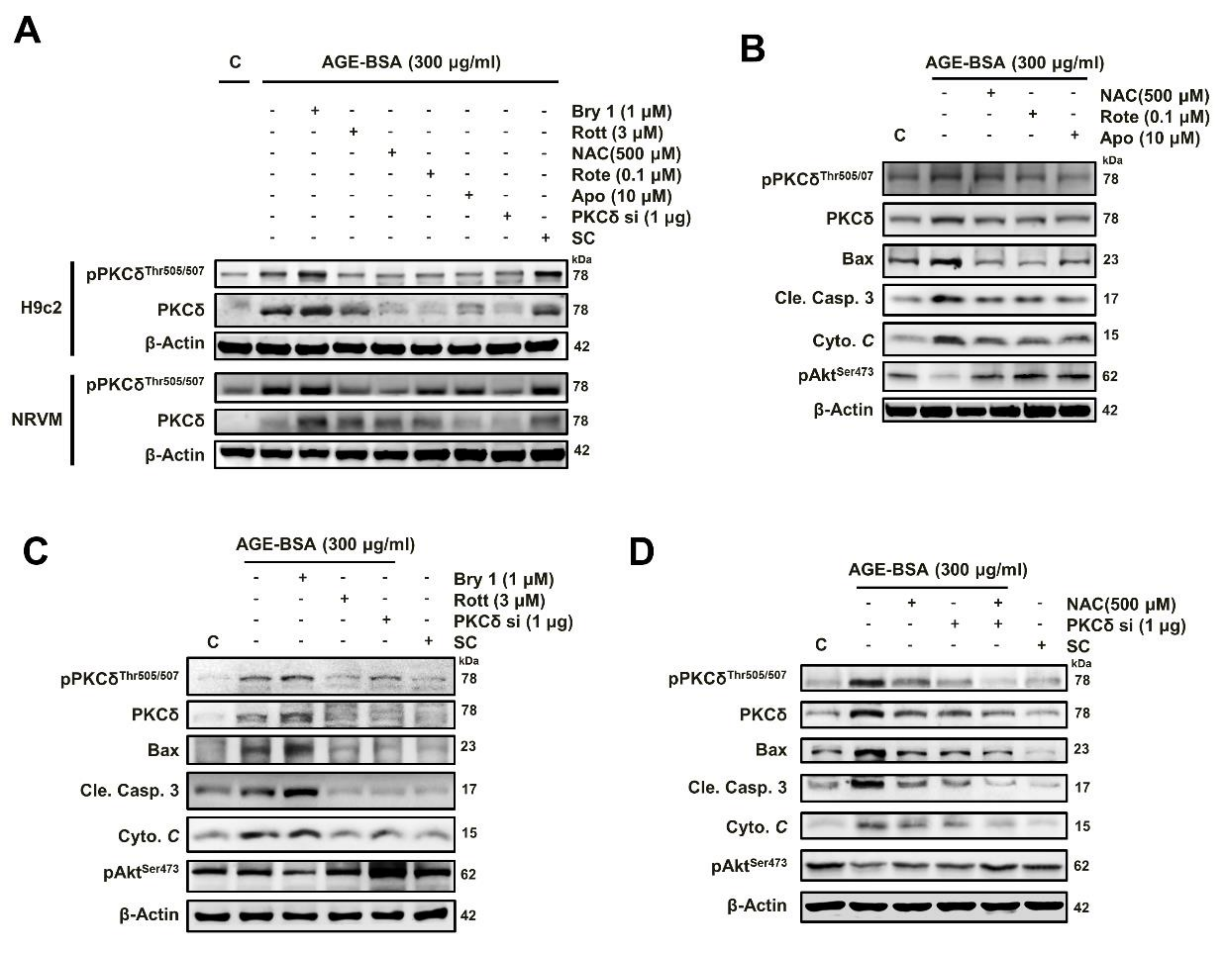
AGE-BSA-induced cardiomyocyte apoptosis is mediated through ROS-dependent PKCδ activation NRVM and H9c2 cells were exposed to AGE-BSA (300 μg/ml) for 24 h. Cells were co-treated with bryostatin 1, a PKCδ activator (100 nM), rottlerin, a PKCδ inhibitor (3 μM), NAC (500 μM), Rote (0.1 μM), Apo (10 μM) or siPKCδ (1 μg). (**A**) Expression and phosphorylation of PKCδ and (**B, C, D**) apoptosis-related proteins were examined by western blot analyses. These are cropped blots, full-length blots of PKCδ and pPKCδ are presented in Suppl. Figure S5. β-Actin was used as a loading control. N-acetylcysteine, NAC; Rote, rotenone; APO, apocynin; SC, scramble.

### AGE-BSA-induced cardiomyocyte apoptosis is through ROS-mediated PKCδ activation

To further examine the potential involvement of PKCδ in the AGE-induced apoptosis, two overexpression plasmids were used: wild type (GFP-PKCδ-WT) and its kinase-dead (KD), catalytically inactive PKCδ^K376R^ (GFP-PKCδ-KD, an ATP binding mutant of PKCδ, lacking kinase activity, as well as this mutant can competitively inhibit the kinase-dependent action of endogenous PKCδ). [31-33] (Fig. 4A). As shown in Fig. 4(B & C) (Suppl. Fig. S4 B&C), increased cytochrome *C* protein level, activations of caspase 3, decreases in the survival protein Bcl2 and Akt phosphorylation induced by AGE exposure were reversed by a PKCδ inhibitor, rottlerin (Rott), or PKCδ siRNA. These results suggest that AGE-BSA-induced apoptosis was associated with PKCδ activation. Therefore, cardiac PKCδ activation by transfection may be used as an AGE exposure model to confirm the effects of AGE on cardiac cells.

As shown in Fig. 4(D) (Suppl. Fig. S4D), the effect of GFP PKCδ-WT overexpression was shown to induce cell apoptosis. As shown in Fig. 4E, at concentrations at 5 μM, rottlerin completely inhibited the cell apoptosis. Plasmids overexpressing GFP-PKCδ-WT or GFP-PKCδ-KD were transfected into cardiac cells. As shown in Fig. 4(F) (Suppl. Fig. S4F), the apoptosis induced by AGE-BSA exposure or overexpression of GFP-PKCδ-WT was not observed in cells with GFP-PKCδ-KD overexpression, indicating the involvement of PKCδ in the AGE-induced cardiac apoptosis.

As shown in Fig. 4(G&H) (Suppl. Fig. S4 G&H), we transfected the cells with GFP-PKCδ-WT and GFP-PKCδ-KD to confirm the AGE-BSA-induced apoptosis through the activation of PKCδ in cardiac cells. Based on the western blot analysis results of fusion protein GFP, its molecular weight shifted from 27 of vector only to 105 of carrying PKCδ vectors, including WT and KD, indicating the successful transfection (Fig 4G). AGE-BSA exposure and GFP-PKCδ-WT overexpression induced apoptosis in both NRVM and H9c2 cells, and these effects were reversed by GFP-PKCδ-KD transfection or the PKCδ inhibitor rottlerin, respectively. These results suggest that AGE-induced apoptosis is mediated through the activation of PKCδ in cardiomyocytes.

**Figure 6.**
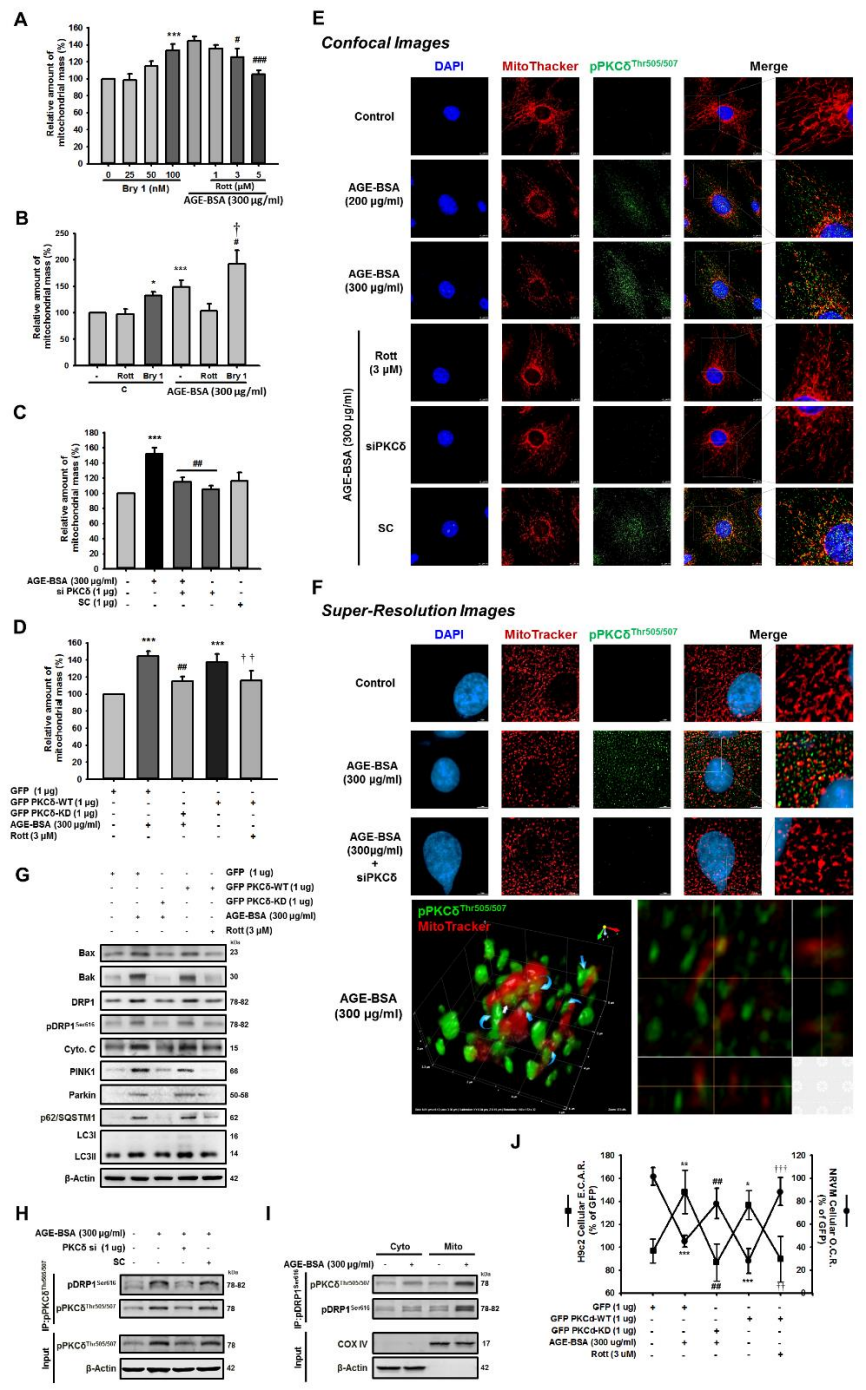
AGE-BSA-induced mitochondria damage and decreased biological function is through PKCδ activation and colocalization in cardiac cells (**A, B**) Cells were treated with the PKCδ activator, bryostatin 1 (100 nM) or inhibitor, rottlerin, (3 μM) for 24 h following exposure to AGE-BSA (300 μg/ml). (**C**) AGE-BSA-exposed cells were transfected with PKCδ siRNA (1 μg) for 24 h. (**D**) Cells were transfected with GFP-PKCδ-WT plus rottlerin or AGE-BSA-exposed cells were transfected with GFP-PKCδ-KD. Mitochondrial damage was evaluated by mitochondrial mass, which was analyzed by flow cytometry. (**E**) AGE-BSA-treated cells were administered the inhibitor rottlerin (3 μM) or siPKCδ for 24 h. The mitochondrial fission and colocalization with PKCδ were examined by immune-fluorescence and analyzed by confocal microscopy. (**F**) Super resolution images of mitochondria structure in cells were analyzed with similar experimental procedures. Blue, DAPI (4, 6-diamidino-2-phenylindole)-stained nuclei; red, MitoTracker Red CMXRos-stained mitochondria. (**G**) The levels of proteins associated with mitochondrial fission and mitophagy in cells were analyzed with similar experimental procedure. These are cropped blots; full-length blots are presented in Suppl. Figure S6. Neonatal rat ventricular myocyte (NRVM) and H9c2 were then analyzed for (**H**) Cells were treated with AGE-BSA for 24?hr and then transfected with the PKCδ siRNA (1 μg) for 24?hr. Cell lysates were immunoprecipitated using antibodies against pPKCδ^T505/507^. Protein expression was detected by immunoblotting. (**I**) The cytosolic and mitochondrial fractions were isolated and subjected to immunoprecipitation followed by western blot analysis. These are cropped blots; full-length blots are presented in Suppl. Figure S7. (**J**) Cellular oxygen consumption rate (O.C.R) and extracellular acidification (E.C.A.R) using an XF24 bioenergetics assay (Seahorse Bioscience, Billerica, MA). Data are expressed as the mean ± SEM, n=3. ^*^P<0.05, ^**^P<0.01, ^***^P<0.001 compared with the control or GFP group, *^#^P*<0.05, ^##^*P*<0.01, ^###^*P*<0.001 compared with the AGE-BSA (300 μg/mg) group, *^†^P*<0.05 compared with the Bry1-treated group, *^††^P*<0.01, *^†††^P*<0.001 compared with the GFP-PKCδ-WT overexpression group.

As shown in Fig. 5 (Suppl. Fig. S5) to confirm that ROS mediates the AGE-induced PKCδ activation to cause cell apoptosis, H9c2 and NRVM cells were treated with AGE-BSA plus the PKCδ activator Bryostatin 1 (Bry1), the inhibitor Rott, siPKCδ or the ROS inhibitors N-acetyl-L-cysteine (NAC), Apocynin (APO) and mitochondrial complex I inhibitor, Rotenone (Rote). Western blotting analyses showed that the AGE-BSA induced PKCδ phosphorylation was ROS-dependent.

### AGE-BSA-induced cardiac mitochondrial dysfunction is mediated through ROS-dependent PKCδ activation and mitochondrial colocalization

Since the unique bioenergetic requirements of the heart, the mitochondria are organized throughout the cardiomyocytes with the largest proportional density. Its function is tightly associated with the power provided for the cardiac action. To demonstrate that PKCδ plays a role in AGE-BSA-induced mitochondrial damage, we examined cells treated with different doses of Bryostatin 1 (Bry1); the results showed a dose-dependent increase in mitochondrial mass. Furthermore, the increased levels by AGE-BSA were dose-dependently reduced by rottlerin (Fig. 6A). These results were confirmed by the findings that the AGE-BSA-induced mitochondrial mass increase was reduced by rottlerin and further enhanced by Bry1 (Fig. 6B). As shown in Fig. 6(C), the increases in mitochondrial mass by AGE-BSA were reduced following siPKCδ transfection. As shown in Fig. 6(D), we confirmed these observations by overexpressing GFP PKCδ wild-type (WT) or its kinase-deficient (KD) mutant in cells. The mitochondrial mass induced by AGE-BSA exposure or GFP PKCδ-WT transfection was reversed by GFP PKCδ-KD transfection or rottlerin, a PKCδ inhibitor, respectively, indicating that the AGE-BSA-induced mitochondrial mass increase is mediated via PKCδ activation.

To further study the role of PKCδ in AGE-BSA cardiotoxicity, mitochondrial fission was examined by immunofluorescence assays. These results were confirmed with fluorescent dye, Mitotracker Red. As shown in Fig. 6(E), in addition to increased PKCδ activation, the administration of high doses of AGE-BSA (200, 300 μg/ml) could induce PKCδ activation and mitochondrial fragmentation. Furthermore, the translocation of activated PKCδ to the mitochondria is generally accompanied by excessive mitochondrial fragmentation, which was inhibited by the rottlerin and siPKCδ treatments, indicating that PKCδ activation was involved in AGE-BSA-induced mitochondrial dysfunction. Super-resolution microscopy images of the mitochondria are shown in Fig. 6(F). Upon activation of PKCδ by AGE-BSA exposure, the mitochondrial network changed its shape with elongation and swell, as well as aggregated fragmented mitochondria were observed and this was inhibited by the treatments of rotterlin and siPKCδ. These data suggest that AGE-BSA-induced mitochondrial dysfunction, such as mitochondrial fission, is through PKCδ colocalization and activation.

Drp1 (dynamin-related protein 1) is a mitochondrial dynamic regulator that promotes mitochondria-mediated constriction to induce mitochondrial fission and apoptosis. Its phosphorylation site, serine 616 (S616), can be phosphorylated by PKCδ, thus increasing mitochondrial fragmentation [18]. During apoptosis, DRP1 with Bax and Bax foci accumulate in the mitochondria and promote substantial mitochondrial fission to further enhance caspase activation [30, 34]. Therefore, Drp1 plays a central role in this process [34-37]. Mitophagy is the selective engulfment of mitochondria by autophagosomes and their subsequent catabolism by lysosomes [38]. It often occurs to defective mitochondria following damage or stress. Previous reports indicated PINK1 and Parkin are involved in the regulation of mitochondrial dynamics [39]. The mitochondrial accumulation of proteins that are poly-ubiquitinated recruits the ubiquitin- and LC3-binding adaptor protein p62/SQSTM1 after Parkin translocation [40]. To assess the effect of AGE-mediated PKCδ activation on cardiac cells, we reduced the enhanced levels of these proteins mediated by PKCδ-WT overexpression and AGE-BSA exposure using rottlerin and PKCδ-KD overexpression, respectively. As shown in Fig. 6(G) (Suppl. Fig. S6), these data suggest that PKCδ is a target of AGE-BSA, which results in abnormal mitochondrial function, initiation of apoptosis and cardiotoxicity.

**Figure 7.**
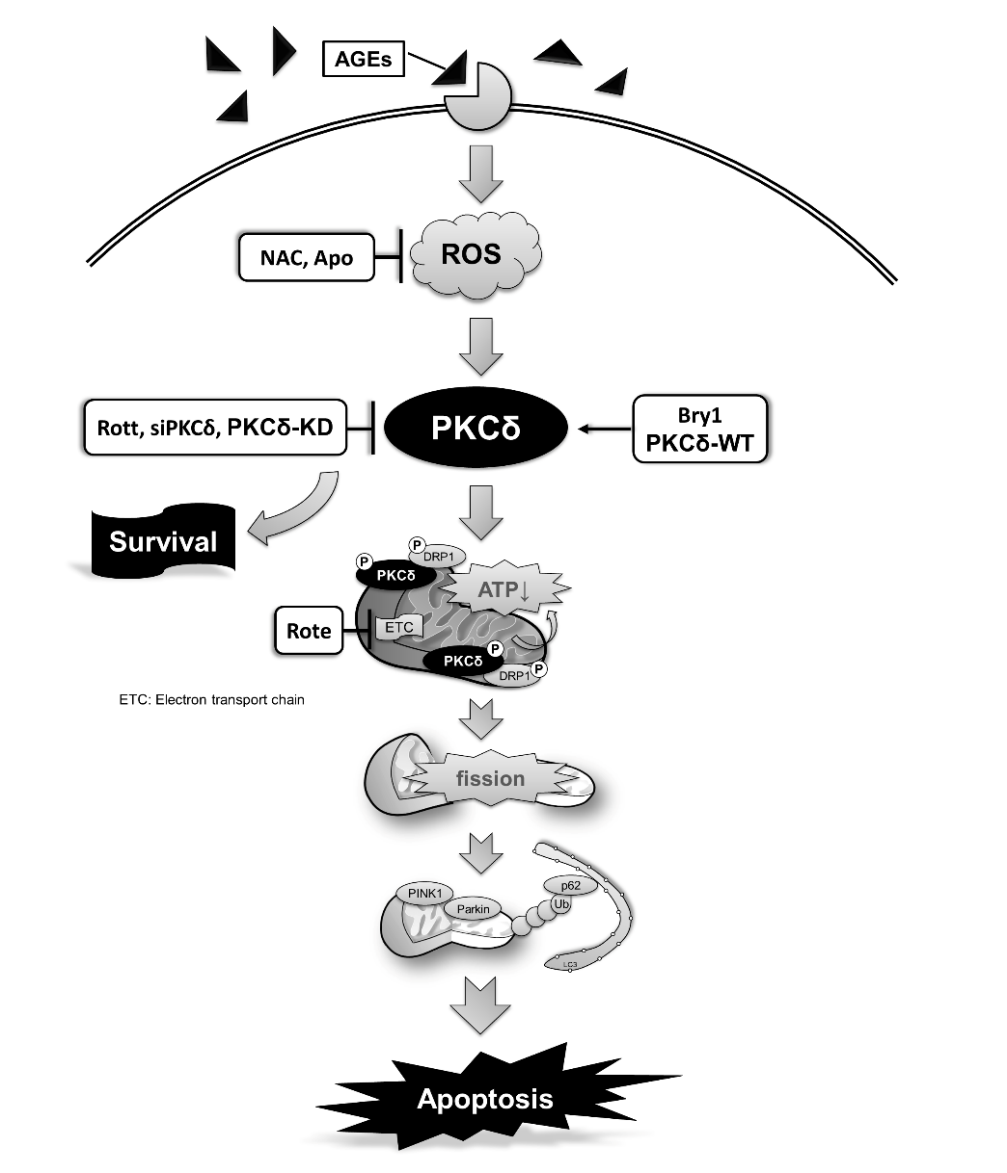
Molecular events of ROS-dependent PKCδ activation involved in the AGE-BSA-induced cardiomyocyte apoptosis. PKCδ activation is involved in the regulation of AGE-BSA-induced cell apoptosis via ROS production and may play a key role in the development of cardiac mitochondrial dysfunction in rats with diabetes or obesity.

Because siPKCδ abolished PKCδ co-localized to the mitochondria (Fig. 6E&F) in cardiomyocytes, we next determined whether Drp1 and PKCδ interact directly. In the previous study, it has been known that Drp1 is phosphorylated by PKCδ and the phospho-Drp1 is formed a complex with PKCδ. The activated PKCδ is translocated to mitochondria through the interaction of the mitochondrial outer membrane protein Fis1 with phospho-Drp1 in neuron suffered oxidative stress [18]. We further study whether the role of the phospho-Drp1 is translocated to the mitochondria and is co-localized with activated PKCδ on the mitochondria in cardiomyocytes. As shown in Fig. 7(H) (Suppl. Fig. S7), we found that siPKCδ treatment reduces PKCd binds to DRP1 protein levels following AGE-BSA exposure, suggesting that AGE-BSA promotes DRP1 protein activated by elevated PKCδ phosphorylated. As shown in Fig. 7(I) (Suppl. Fig. S7), the cytosolic and mitochondrial fractions were isolated and subjected to immunoprecipitation further revealed that ectopic PKCδ was associated with DRP1. These results suggested that the phospho-Drp1/phospho-PKCδ complex is enriched in the isolated mitochondria.

To assess AGE-BSA-induced changes in the mitochondria function of cardiomyocytes, metabolic capacity and extracellular acidification were evaluated. Neonatal ventricular cardiomyocytes were treated with AGE-BSA at 300 μg/ml for 24 h or PKCδ-WT transfection, followed by PKCδ-KD overexpression or rottlerin, respectively. Then, we measured the rates of cellular oxygen consumption (OCR) and extracellular acidification (ECAR) using a Seahorse XF24 flux analyzer. As shown in Fig. 7(J), the data from both AGE-BSA-exposure and PKCδ WT overexpression were similar, and the decreased cellar OCR and increased ECAR following AGE-BSA exposure and PKCδ WT overexpression were reversed by PKCδ-KD transfection and rottlerin treatment, respectively, suggesting that PKCδ regulates mitochondrial energy and redox metabolism in cardiomyocytes exposed to AGE-BSA. These findings indicate that the AGE-BSA-induced mitochondrial biological dysfunction is mediated through PKCδ activation.

### DISCUSSION

DM is one of the leading causes of death worldwide. Hyperglycemia is considered the principal cause of diabetic complications [7]. The increased level of blood glucose enhances the formation of sugar-derived circulatory substances called AGEs. AGEs production and accumulation are often associated with an increase of ROS and linked to multiple sequelae, such as diabetic cardiomyopathy [41, 42]. Therefore, the effects of AGE accumulation may have an impact with heart failure. In this study, we investigated the role of PKCδ signaling in regulating cardiomyocyte apoptosis in response to AGEs in both cell and animal models. Our results (Fig. 7) demonstrate that the ROS-dependent PKCδ activation by AGE-BSA triggered mitochondrial dysfunction and further led to cardiac cell death, supporting the hypothesis that PKCδ exerts as an important regulator of mitochondrial function and apoptosis in cardiomyocytes following AGE-BSA exposure.

PKCδ, a member of a novel class of protein kinase C, contains amino-terminal regulatory and carboxyl-terminal catalytic domains separated by a flexible hinge region. PKCδ is shown to regulate a variety of biological functions in different cell types, including proliferation, survival, and apoptosis. PKCδ is also reported to involve invasion and metastasis in cancer cells [43-45]. Notably, recent studies suggest PKCδ mediated the regulation of mitochondrial fission in neuropathogenesis [18, 31]. PKCδ is activated for phosphorylation by a variety of stimuli [11], such as ROS, anti-cancer agents, ultraviolet radiation [46], growth factors, and cytokines [47]. There are several phosphorylation sites in PKCδ modulated in a cell type and stimulus-specific manner. The phosphorylation of different sites contributions to different results of PKCδ activation[48]. In addition, the threonine phosphorylation in the activation loop segment of C-terminal is required for its kinase activity [13, 49]. Among the stimuli, the H_2_O_2_ treatment in human cells, tyrosine and threonine phosphorylations are observed to result in mitochondrial dysfunction, and threonine 505 has also been detected in response to H_2_O_2_-induced apoptosis [48, 50]. The phosphorylation at catalytic fragment demonstrated the result of cell death which is associated with the caspase-3-dependent cleavage of PKCδ. This is in consistent with our results (Fig. 4F) that overexpression of PKCδ-KD, which is mutated at PKCδ^K376R^ to cause catalytical inactivation, resulted in the inhibition of apoptosis. Furthermore, translocation of PKCδ to the mitochondria, cytoplasm, nucleus and other cellular organelles after phosphorylation initiates programmed cell death [11, 14, 51-54]. In our study, phosphorylation of PKCδ at Threonine 505 was observed in cells and colocalization with mitochondria following AGE exposure. At the same time, cell apoptosis and mitochondrial dysfunction were also observed. Additionally, using AGE exposure to investigate the involvement of PKCδ, we also set up a PKCδ-WT overexpression model to represent AGE effect, and rottlerin was applied as a PKCδ inhibitor which has been shown to have many off-target effects. However, the selections for PKCδ inhibitory action in the present study include siRNA and PKCδ-KD overexpression (with a dominant negative activity). The treatments of PKCδ siRNA showed the same results in experiments, such as Fig. 4C, Fig. 5(A&C), Fig. 6C and the images in Fig. 6(E&F), and the treatments of PKCδ-KD overexpression showed the similar results in Fig. 4(G&H), Fig. 6(D&G&H). Therefore, the off-target property of rottlerin is not able to limit the conclusion being made.

Mitochondria provide cellular energy and are critical for cell survival; its morphology is regulated by a balance between fission and fusion which is associated with the development of apoptosis [18, 19]. The quality control by mitophagy is the distinction between damaged and healthy mitochondria. In our study, using the antibody of phosphorylated PKCδ and phosphorylated Drp-1 to detect the effect of PKCδ activation on mitochondrial morphology, the data demonstrated that PKCδ-induced mitochondrial fragmentation may be associated with a disruption of mitochondrial fusion. And mitophagy is regulated by PINK1 and parkin proteins; the parkin recruitment to depolarized mitochondria promotes their degradation. Autophagy adaptor p62/SQSTM1 might also be involved in mitophagy as it has ubiquitin- and LC3- binding domain and has a role in recruiting autophagosomes to ubiquitylated protein aggregates [38, 39]. Indeed, it was observed that treatment with a PKCδ inhibitor or PKCδ-KD reduced Drp1 phosphorylation induced by PKCδ-WT overexpression or AGE-BSA exposure, respectively (Fig. 6G), supporting our hypothesis that AGE-BSA-induced Drp1 phosphorylation is likely to be mediated through PKCδ activation. In addition to the selective removal of damaged mitochondria, mitophagy might also require regulating mitochondrial numbers to changing cellular metabolic needs.

Furthermore, ligand engagement of RAGE by AGEs results in the production of cellular ROS [55]. A previous study [56] showed that high glucose-induced cellular ROS generation in human peritoneal mesothelial cells (HPMC) via activation of nicotinamide adenine dinucleotide phosphate (NADPH) oxidase, reducing the rate of mitochondrial metabolism, suggesting that ROS and mitochondrial dysfunction are important pathogenetic mediators of diabetic complications. In Lee’s study [20], the increase of mitochondrial mass and intracellular ROS levels accompanied by up-regulation of PKCδ phosphorylation was observed in human osteosarcoma 143B cells following H_2_O_2_ treatment. Therefore, PKCδ may play a role in the H_2_O_2_-induced increase of mitochondrial mass, which was completely inhibited by a PKCδ inhibitor, rottlerin. These data indicate that mitochondrial targeting of PKCδ is required for the response of cells to oxidative stress. Moreover, it is also reported that PKCδ activity was increased by phosphorylation of threonine 505/507 and tyrosine 313/311 in response to oxidative stress [20, 48, 57]. Another study [58] indicated that phosphorylation of T505 in the activation loop in the hydrophobic C-terminus appears to be important for PKCδ activation. Additionally, activation of PKCδ is required for its mitochondrial localization and resulted in cytochrome *c* release, caspase activation, and cardiac apoptosis [14, 15, 20]. In our study, firstly, the results of the western blot analyses and IHC staining using PKCδ T505/507 phosphorylation antibodies showed PKCδ activation in the hearts of animals with DM and a high-fat diet. The analysis of Seahorse XF24 flux analyzer demonstrated that AGE-BSA treatment reduced mitochondrial functions, which were reversed by the PKCδ inhibitor. Secondly, super-resolution microscopy images clearly indicated that the colocalization of activated PKCδ and mitochondria, and AGE exposure-induced mitochondrial fission were ameliorated by the PKCδ siRNA treatment. Therefore, our evidence strongly supports the hypothesis that AGEs enhanced PKCδ activation and its colocalization to mitochondria, further promoting mitochondrial fission, and reducing its biological function which results in cell death, leading to heart failure.

The heart is an organ requesting the highest amount of ATP. It predominantly relies on mitochondrial metabolism to provide the energy needed to pump blood to entire body. In addition, the physiology and oxidative stress-induced cellular damage from mitochondria are considered to constitute a central event in apoptosis [19]. Therefore, heart function is closely related to the mitochondrial action. The failure of mitochondria function contributes to the development of cardiac failure. On the other hand, AGEs have been shown to impair respiratory function and overproduction of ROS, which facilitates the production of mitochondrial superoxide contributing to tissue damage in diabetic patients [10, 59, 60]. Here, we provide the evidence of the involvement of mitochondrial dysfunction and oxidative stress in the cellular toxicity produced by AGE-BSA. Our findings demonstrated that PKCδ is a critical regulator of mitochondrial fission in diabetic cardiomyocytes. We conclude that cardiac ROS-dependent PKCδ activation can induce its colocalization with mitochondria, Drp1-dependent mitochondria fission and fragmentation and reduced biological functions resulting in cardiac failure. Therefore, the development of new therapeutic strategies that is capable of preventing AGE induced-ROS-dependent PKCδ activation may be a promising treatment for cardiomyopathy caused by increased levels of AGEs.

## Supplemental data

Supplemental data are available at www.aginganddisease.org/EN/10.14336/AD.2017.0924
