# Supplementary material for: Pkcδ Activation is Involved in ROS-Mediated Mitochondrial Dysfunction and Apoptosis in Cardiomyocytes Exposed to Advanced Glycation End Products (Ages)
Source: Aging Dis. 2018 Aug 1;9(4):647–63. doi: 10.14336/AD.2017.0924 (PMC6065295; doi:10.14336/AD.2017.0924)
Supplement: Supplementary file 1 — Supplemental data are available at www.aginganddisease.org/EN/10.14336/AD.2017.0924 [file AD-9-4-647-s1.pdf]

Original Article

Pkcδ Activation is Involved in ROS-Mediated Mitochondrial Dysfunction and Apoptosis in Cardiomyocytes Exposed to Advanced Glycation End Products (Ages)

Yao-Chih Yang[1], Cheng-Yen Tsai[2,3], Chien-Lin Chen[4], Chia-Hua Kuo[5,6], Chien-Wen Hou[5], Shi-Yann Cheng[7,8,9], Ritu Aneja[10], Chih-Yang Huang[11,#], Wei-Wen Kuo[1,#,*]

[1]Department of Biological Science and Technology, College of Biopharmaceutical and Food Sciences, China Medical University, Taiwan. [2]Department of Pediatrics, China Medical University Beigang Hospital, Taiwan. [3]School of Chinese Medicine, College of Chinese Medicine, China Medical University, Taiwan. [4]Department of Life Sciences, National Chung Hsing University, Taiwan. [5]Laboratory of Exercise Biochemistry, University of Taipei, Taipei, Taiwan. [6]Graduate Institute of Physical Therapy and Rehabilitation Science, China Medical University, Taiwan. [7]Department of Medical Education and Research and Department of Obstetrics and Gynecology, China Medical University Beigang Hospital, Taiwan. [8]Department of Obstetrics and Gynecology, China Medical University An Nan Hospital, Taiwan. [9]Obstetrics and Gynecology, School of Medicine, China Medical University, Taichung, Taiwan. [10]Department of Biology, Georgia State University, Atlanta, GA 30303, USA. [11]Graduate Institute of Basic Medical Science, China Medical University, Taichung, Taiwan; Graduate Institute of Chinese Medical Science, School of Chinese Medicine, China Medical University, Taichung, Taiwan; Department of Health and Nutrition Biotechnology, Asia University, Taichung, Taiwan.

© 2017. Yang YC et al. Published online at http://dx.doi.org/10.14336/AD.2017.0924

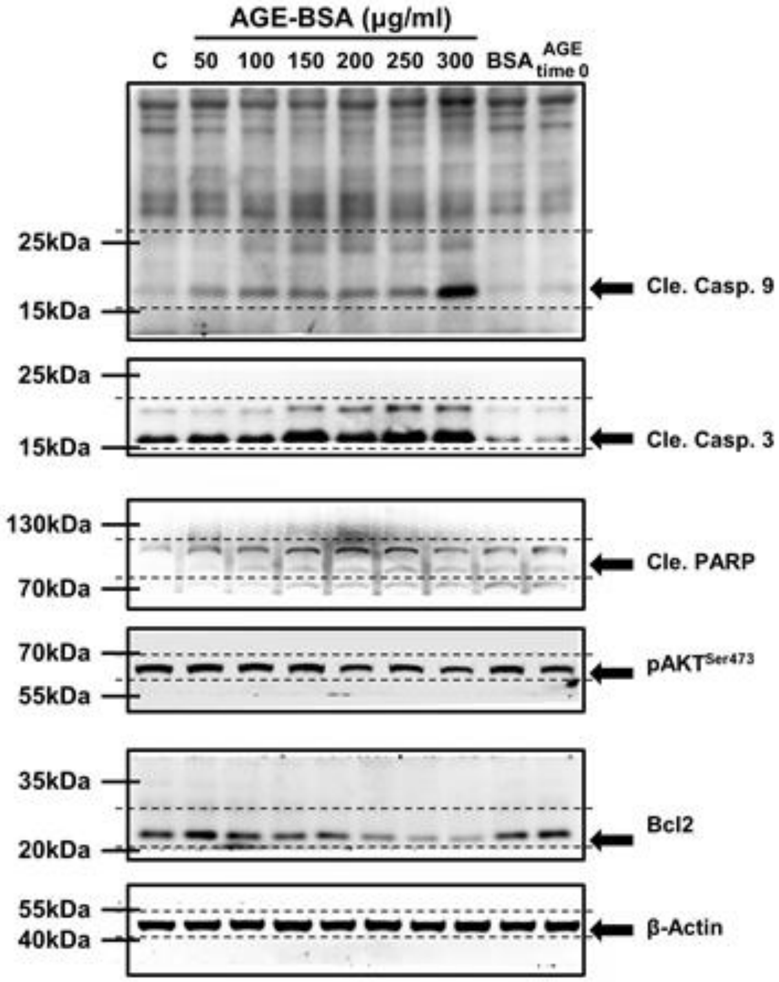

Supplementary Figure 1. Full length blots of cropped images shown in Figure 1B. Caspases are cysteine proteases and are the executioners of apoptosis. Once initiator caspases are activated, they produce a chain reaction, activating several other executioner caspases. Caspase-3 and Caspase-9 shares many of the typical characteristics common to all currently-known caspases. Caspase-9 is formed from a 51/40/38 kDa zymogen that is cleaved into 38/17 kDa subunits. Caspase-3 is formed from a 35 kDa zymogen that is cleaved into 19/17 kDa subunits. PARP is formed from a 116 kDa zymogen that is cleaved into 89 kDa subunits.

© 2017. Yang YC et al. Published online at http://dx.doi.org/10.14336/AD.2017.0924

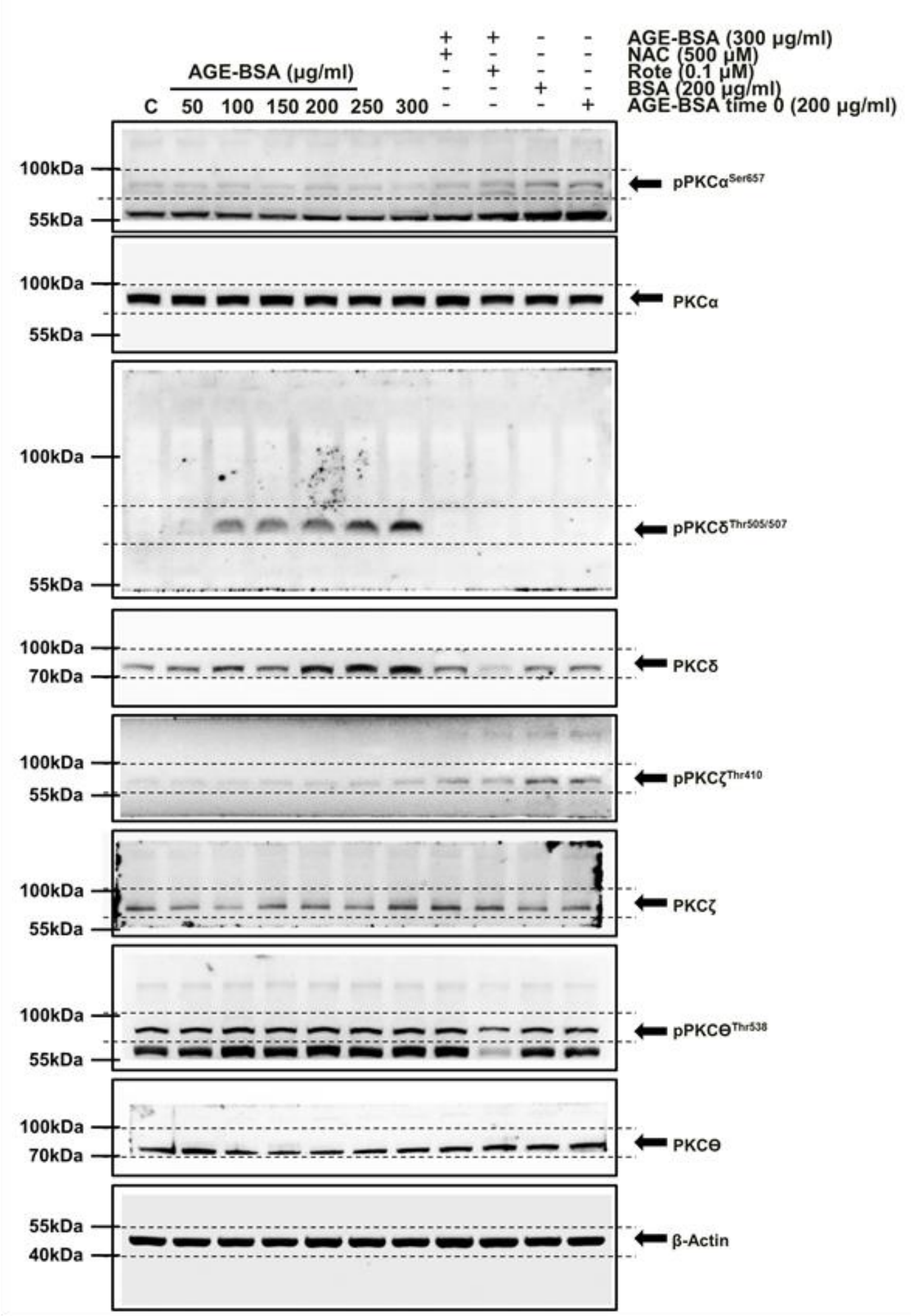

Supplementary Figure 2. Full length blots of cropped images shown in Figure 2A. Western blot analysis was performed with antibodies against the PKC isoforms. PKC isoforms are subdivided into 3 groups based on their lipid and cofactor requirements:

1 . the diacylglycerol (DAG) and calcium sensitive conventional isoforms (α, βI, βII, and γ),
2 . the DAG-sensitive and calcium-insensitive novel isoforms (δ, η, θ, and ε), and
3 . the phosphatidylinositide trisphosphatesensitive atypical isoforms (ζ, ι, μ, and λ).

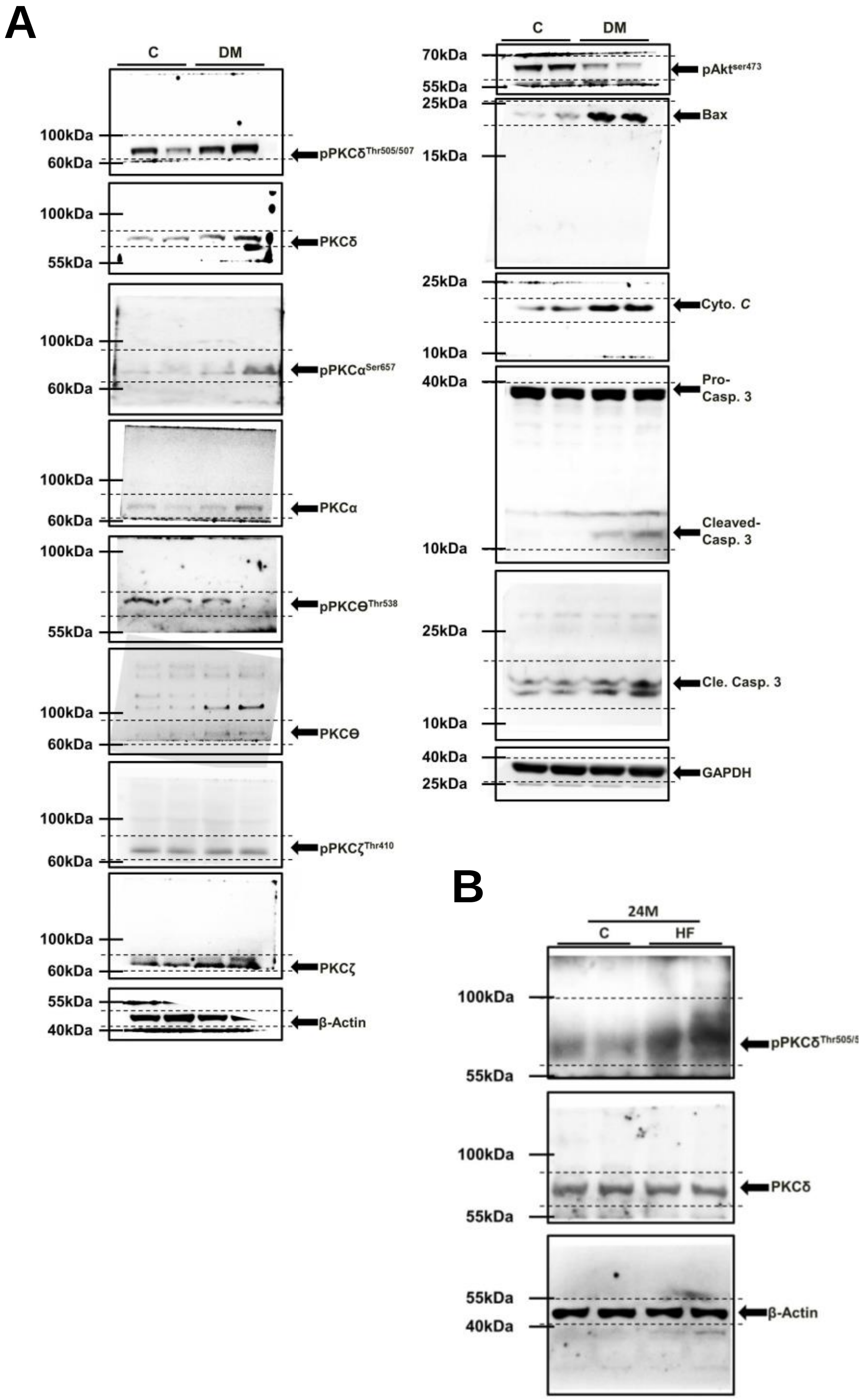

Supplementary Figure 3. Full length blots of cropped images shown in Figure 3A&B. To further identify which PKCs was involved in the mechanism behind diabetes and aging high fat diet -induced cardiac apoptosis, we detected the PKCδ rather than other PKC isoforms from the diabetic and aging high diet rat hearts. Western blot analysis of the cardiac expression and phosphorylation levels of PKC isoforms and apoptosis pathway proteins in rat with (A) diabetes mellitus and (B) aging high-fat diet.

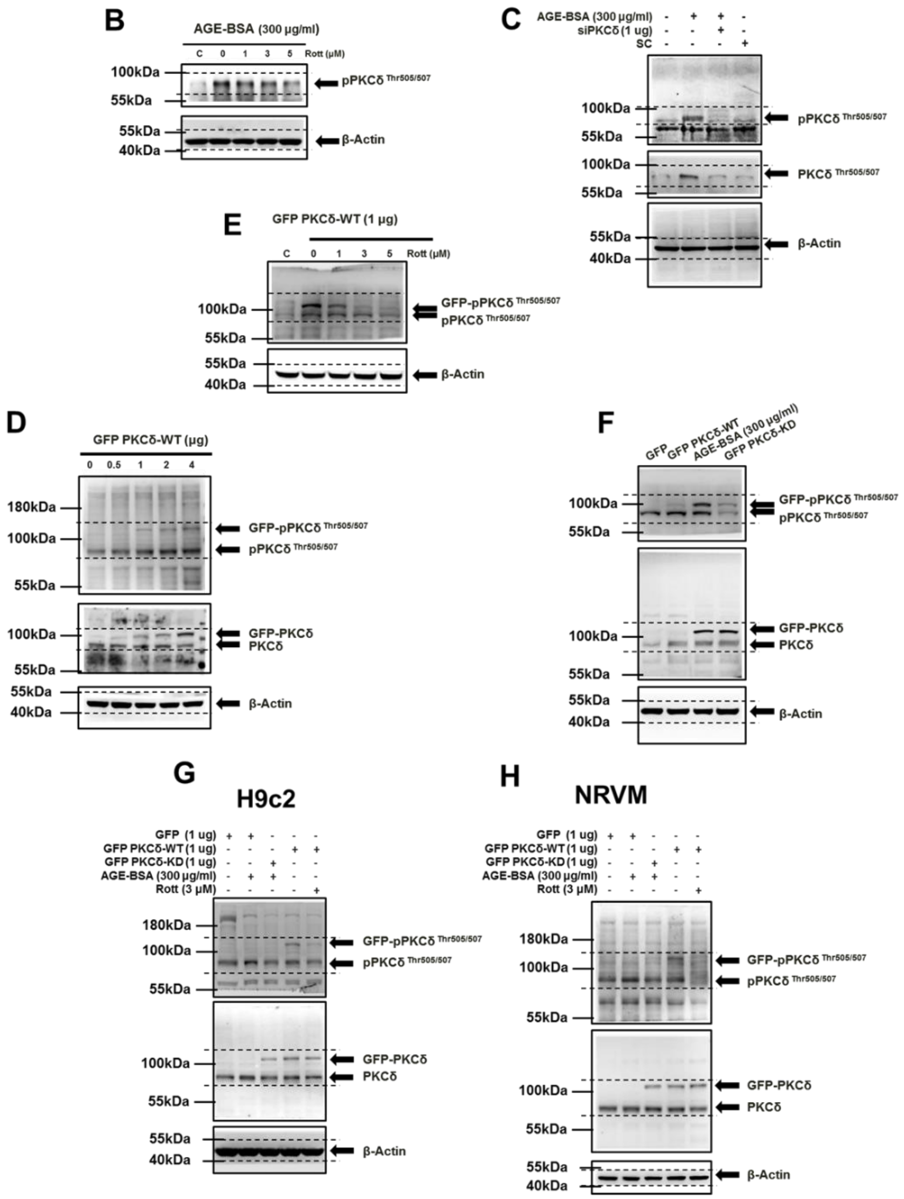

Supplementary Figure 4. Full length blots of cropped images shown in Figure 4(B-H). To examine the potential involvement of PKCδ in the AGE-induced apoptosis, the effect of the selective PKCδ inhibitor, rottlerin, on cell apoptosis was evaluated. (B&C) AGE-BSA significantly increased the levels of phosphorylated PKCδ. (D) The effect of GFP-fused PKCδ (GFP PKCδ-WT) overexpression on the apoptosis of H9c2 cells was measured. Subsequently, an overexpression strategy was used to examine the role of PKCδ in cell apoptosis. (E) At concentrations at 5 μM, rottlerin completely inhibited the cell apoptosis. (GFP PKCδ-WT) and its kinase-deficient (GFP PKCδ-KD) mutant were overexpressed in H9c2 cells. (F) Overexpression of GFP PKCδ-WT or its GFP PKCδ-KD mutant in cells not caused their apoptosis. These results, together suggest that PKCδ may be involved in the AGE-induced cell apoptosis. (G&H) Transfected the cells with (GFP PKCδ-WT) and kinase-deficient (GFP PKCδ-KD) to confirm the AGE-BSA-induced apoptosis through the activation of PKCδ in cardiac cells.

© 2017. Yang YC et al. Published online at http://dx.doi.org/10.14336/AD.2017.0924

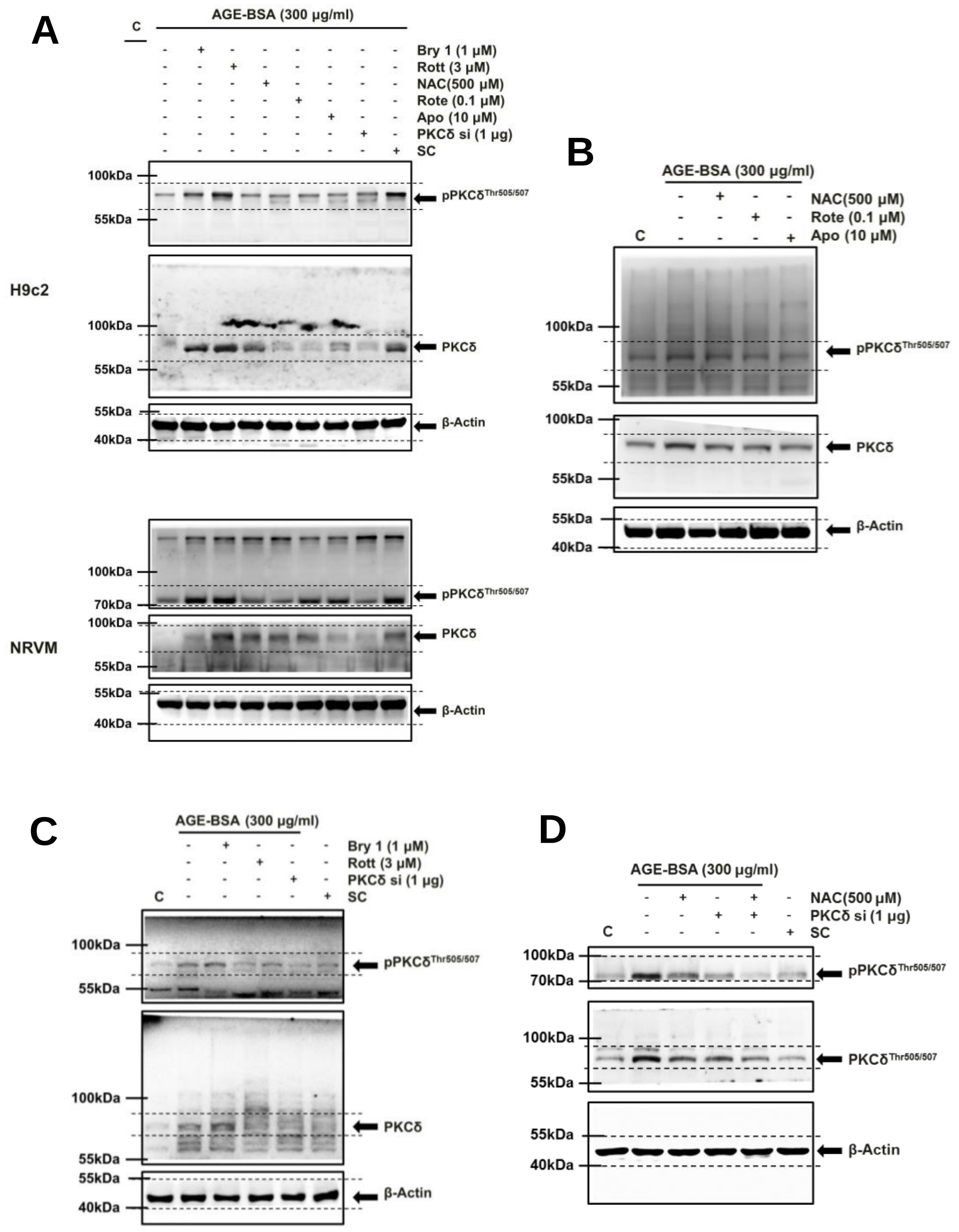

Supplementary Figure 5. Full length blots of cropped images shown in Figure 5. In order to confirm that reduction of ROS generation mediates the anti-apoptotic effects of ROS inhibitor on AGE-BSA induced cell death, H9c2 and Neonatal Rat Ventricular Myocytes (NRVM) were co-treated with AGE-BSA plus PKCδ activator Bryostatin 1 (Bry1), inhibitor rottlerin (Rott), siPKCδ or ROS inhibitor N-acetyl-L-cysteine (NAC), rotenone (Rote) and Apocynin (APO), the western blotting analysis results showed that the AGE-BSA induced PKCδ phosphorylation.

© 2017. Yang YC et al. Published online at http://dx.doi.org/10.14336/AD.2017.0924

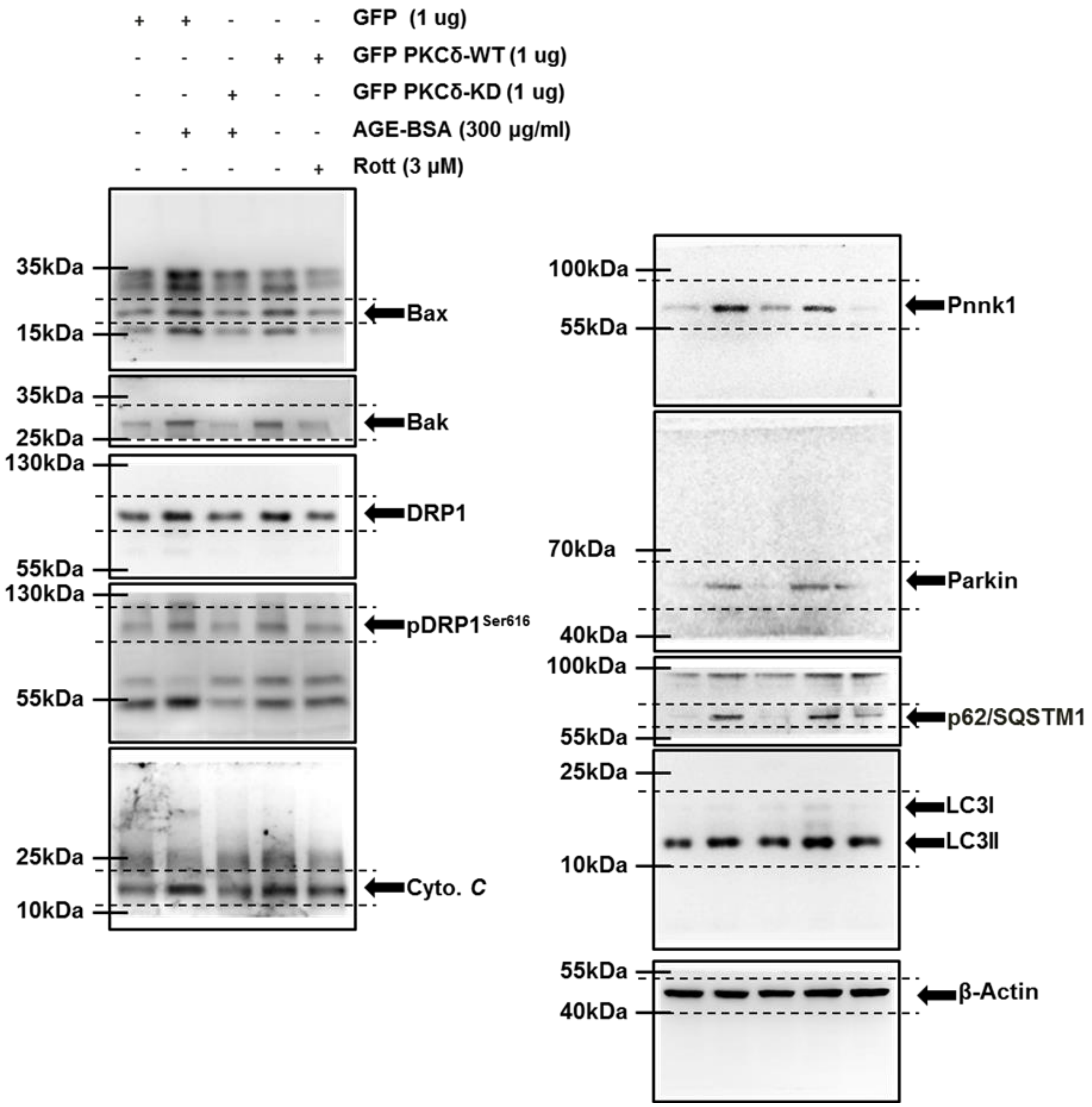

Supplementary Figure 6. Full length blots of cropped images shown in Figure 6G. Drp1 (dynamin-related protein 1) is a mitochondrial dynamic regulator that promotes mitochondria-mediated constriction to induced mitochondrial fission and apoptosis. Its phosphorylation site, serine 616 (S616), can be phosphorylated by PKCδ, thus increasing mitochondrial fragmentation. During apoptosis, DRP1 with Bax and Bax foci accumulates on mitochondrial and mediates dramatic mitochondrial fission to further implicate caspase activation. The pro-apoptotic factors Bax, Bak and cytochrome *c* are released through Bax-lined pores in the outer membrane of Drp1, resulting in mitochondrial fission during apoptosis. PINK1 and Parkin are involved in the regulation of mitochondrial dynamics. The mitochondrial accumulation of proteins that are poly-ubiquitinated recruits the ubiquitin- and LC3-binding adaptor protein p62/SQSTM1 after Parkin translocation.

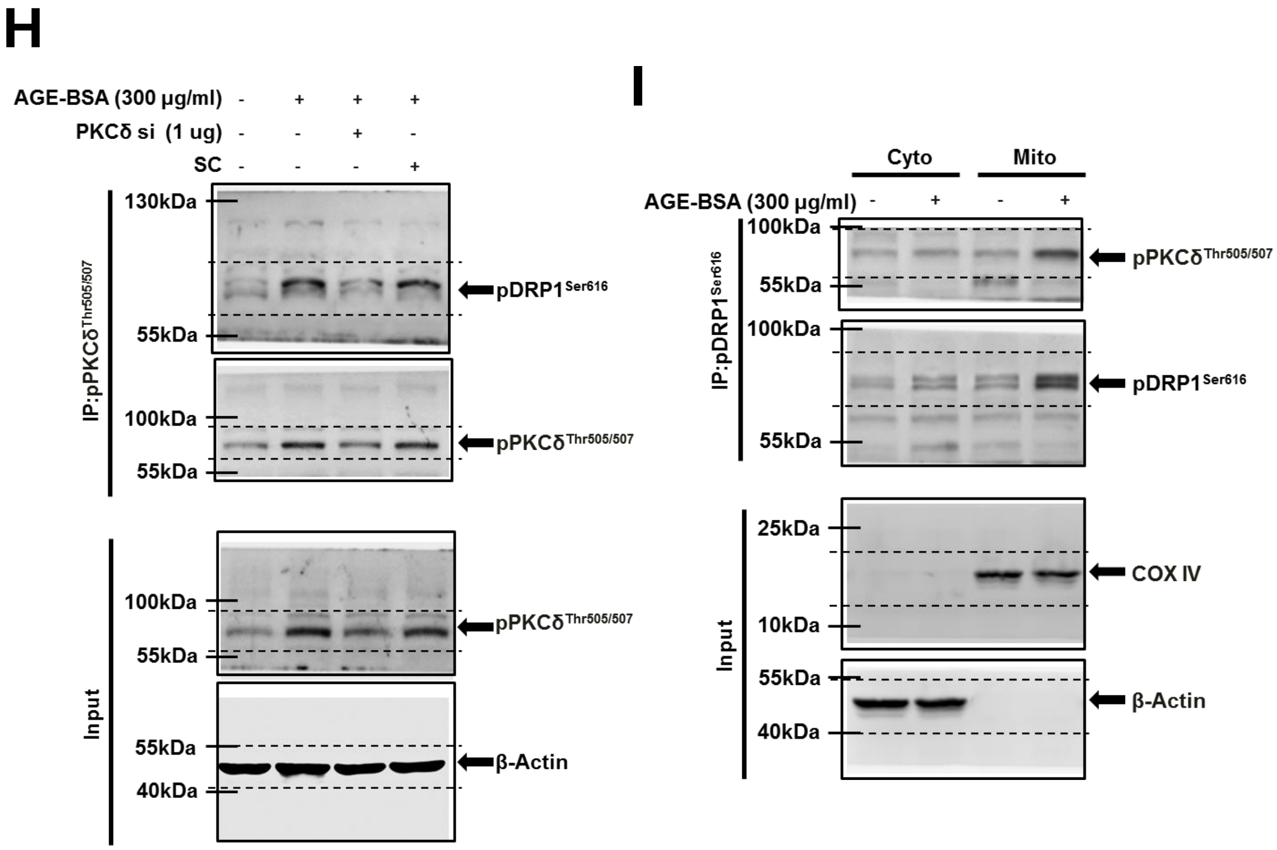

Supplementary Figure 7. Full length blots of cropped images shown in Figure 6 (H&I). The phospho-Drp1 is translocated to the mitochondria and is co-localized with activated PKCδ on the mitochondria. (H) Cells were treated with AGE-BSA for 24 hr and then transfected with the PKCδ siRNA (1 µg) for 24 hr. Cell lysates were immunoprecipitated using antibodies against pPKCδT505/507. Protein expression was detected by immunoblotting. (I) The cytosolic and mitochondrial fractions were isolated and subjected to immunoprecipitation followed by western blot analysis. Control for protein loading was confirmed by cyclooxygenase IV (Cox IV) and β-Actin Western blot.

© 2017. Yang YC et al. Published online at http://dx.doi.org/10.14336/AD.2017.0924

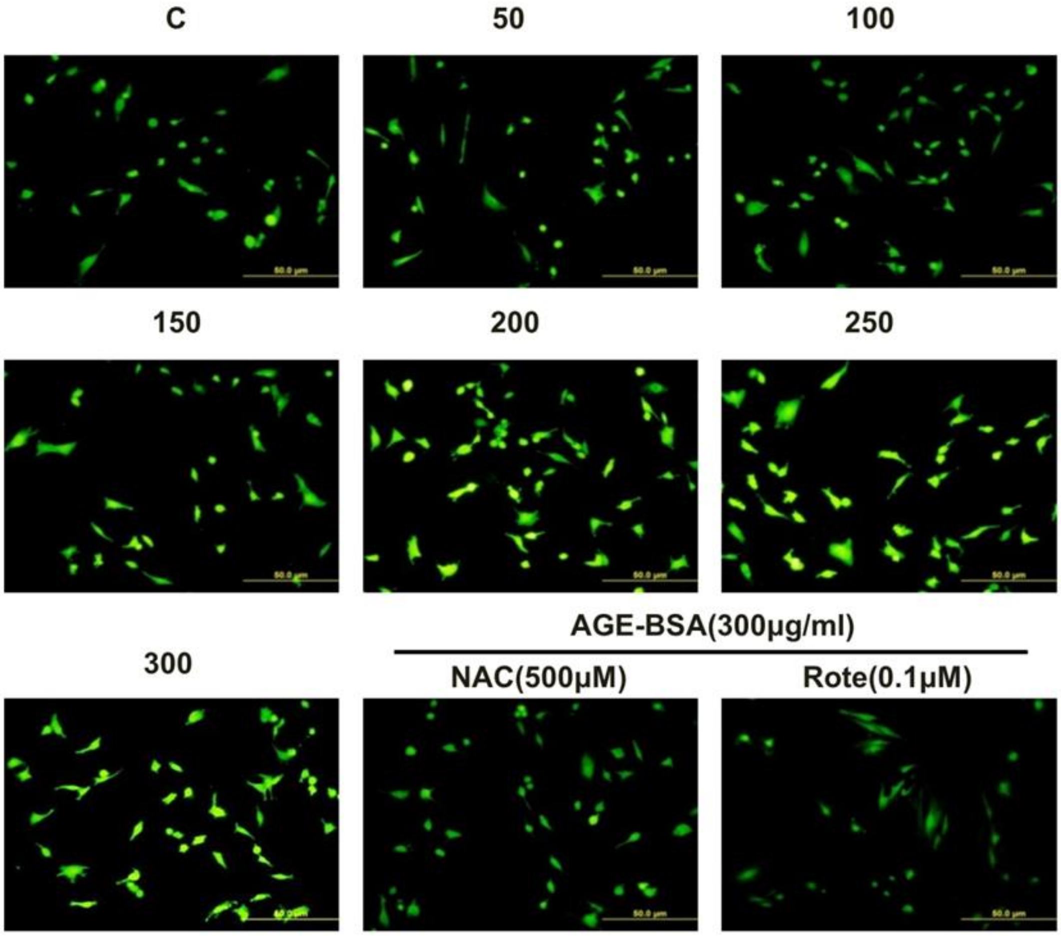

Supplementary Figure 8. AGE-BSA increased intracellular ROS generation in H9c2 cells. To examine the ROS production, the mean fluorescence intensities (MFIs) were measured using 2′,7′-Dichlorofluorescin diacetate (DCFH-DA). Cardiac cells exposed to 0-300 µg/ml AGE-modified BSA (AGE-BSA) dose-dependently increased cellular ROS production. ROS production was attenuated by an ROS scavenger, NAC (500 µM), and the mitochondrial complex I inhibitor Rote (0.1 µM).

© 2017. Yang YC et al. Published online at http://dx.doi.org/10.14336/AD.2017.0924

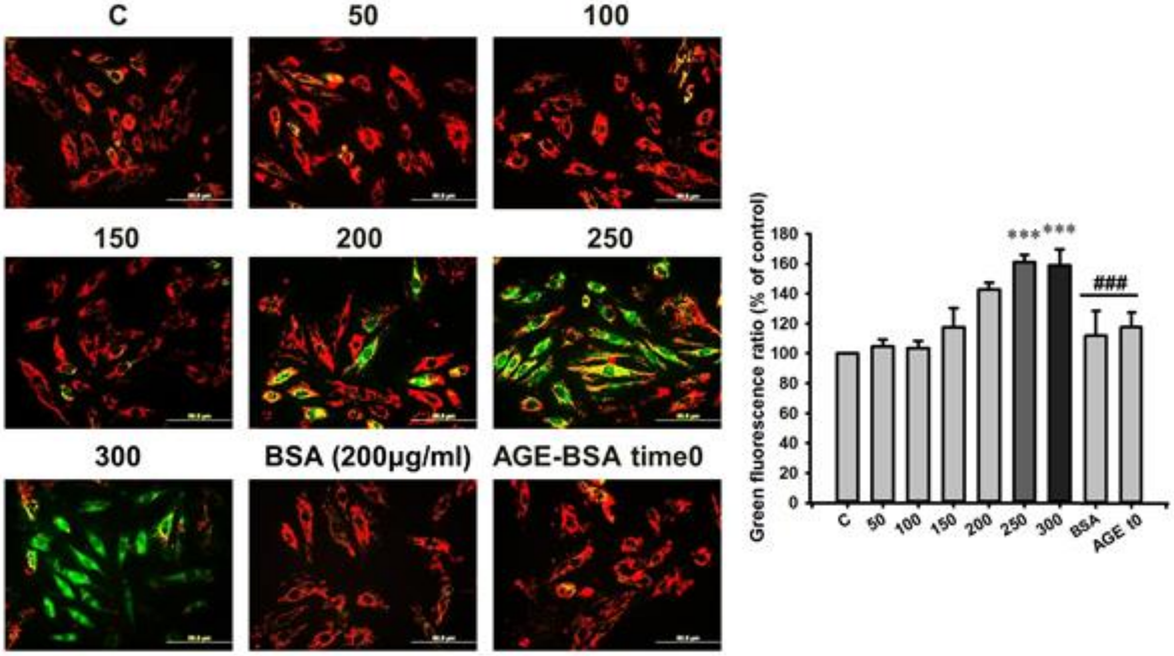

Supplementary Figure 9. AGE-BSA induced mitochondrion damage in cardiac cells. The unique bioenergetics requirements of the heart dictate that not only it generates and maintains the largest proportional density of mitochondria among the organs in the body, but the mitochondria are organized throughout the cardiac myocytes in a highly structured and stable manner. Therefore, to further explore AGE-BSA–induced mitochondrial dysfunction, we performed the fluorescence analysis to examine the changes in mitochondrial membrane potential (Δψm). The results of mitochondrial membrane potential assessed by fluorescence microscope using JC1 dye following the treatment of AGE-BSA. The mitochondrial membrane instability was dose-dependently induced by AGE-BSA treatment. Data are expressed as the mean ± SEM, n=3. $^{***}P$<0.001 compared with the control group, $^{\#\#\#}P$<0.001 compared with the 300 μg/mg group.

JC-1 Staining:

Mitochondrial inner trans-membrane potential (ΔΨm) was assessed using the polychromatic (5, 5′, 6, 6′-tetrachloro-1, 1′, 3, 3′-tetraethylbenzimidazolylcarbocyanine iodide, JC-1; Sigma, Saint Louis, USA). JC-1 is a lipophilic fluorescent cation that can be incorporated into the mitochondrial membrane, where it can form dependent on the membrane potential state aggregates. This aggregation changes the fluorescence properties of JC-1 leading to a shift from green to orange fluorescence. Intact living cells stained with JC-1 exhibited pronounced orange mitochondria fluorescence detectable by confocal microscopy. Apoptosis results in a breakdown of the mitochondrial membrane potential and a subsequent decrease in the orange fluorescence (and a slight increase in the green fluorescence). Therefore, apoptotic cells can be easily distinguished from non-apoptotic cells. In brief, after treatments, cells were washed with PBS and incubated with medium containing JC-1 staining reagent at 37 °C for 20 min followed by washing with PBS. Mitochondrial membrane potential was detected using microscopy.

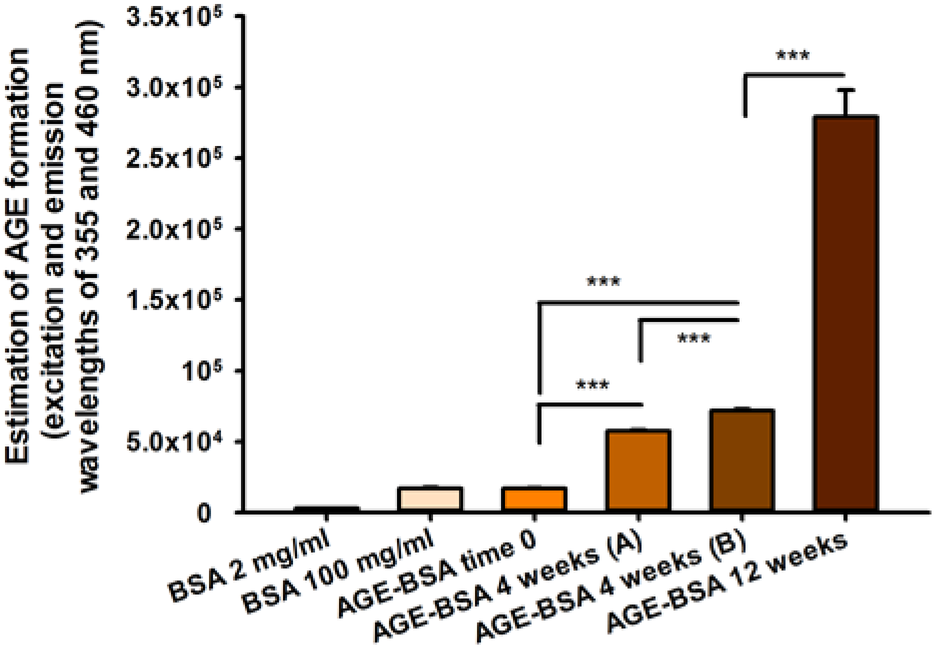

Supplementary Figure 10. The degree of AGE modification of these proteins was determined by competitive AGE-ELISA.
